# An investigation of the effect of the protein corona on the cellular uptake of nanoliposomes under flow conditions using quartz crystal microgravimetry with dissipation†

**DOI:** 10.1039/d4na00783b

**Published:** 2024-10-30

**Authors:** Nicholas Van der Sanden, Radu A. Paun, Michael Y. Yitayew, Oscar Boyadjian, Maryam Tabrizian

**Affiliations:** a Department of Biomedical Engineering, McGill University Duff Medical Building, 3775 University Street Montreal Quebec H3A 2B4 Canada maryam.tabrizian@mcgill.ca; b Faculty of Dental Medicine and Oral Health Sciences, McGill University Montreal Canada

## Abstract

When nanoparticle delivery systems are immersed in biological fluids, a complex assembly of proteins forms on their surface, creating a protein corona. The protein corona alters the physicochemical properties, toxicity, biodistribution, cellular uptake, and immune response of the nanoparticles, and consequently, their therapeutic efficacy. Currently, there is a lack of *in vitro* methods to assess the effects of the protein corona on nanoparticle uptake under dynamic flow and assess their binding kinetics in real-time. Here, we introduce quartz crystal microbalance with dissipation (QCM-D) as an *in vitro* technique, capable of incorporating dynamic flow, to study the effect of the protein corona on the binding of nanoliposome (NLP) formulations to cell surfaces as a first step in their cellular uptake. The interactions of four NLP formulations (low PEGylated, high PEGylated, negatively charged and positively charged NLPs) with A375 melanoma and THP1 cell lines were assessed by QCM-D, before and after the formation of a protein corona. Through real-time recording of the frequency and dissipation shifts (Δ*f* and Δ*D*, respectively), the QCM-D results provided strong evidence of the role of the protein corona in the cellular interaction of these NLP formulations, with a variation in their adsorption kinetics depending on their initial composition. NLP's attachment to the cell surface was the lowest for PEGylated NLPs (<5%), while the positively charged NLPs showed the highest cellular attachment (≈100%), regardless of the presence of the protein corona or cell type. The effect of the protein corona was more pronounced for the negatively charged NLPs, where a significant reduction in the NLP attachment was observed. To complement the QCM-D data on the NLP attachment and to determine whether the NLP attachment leads to cellular uptake, confocal microscopy and flow cytometry were used to confirm NLP uptake by A375 and THP1 cells. Proteomic analysis revealed a differential composition of the protein corona on the various NLPs with possible implications for their sequestration and cellular uptake. Collectively, the findings suggest that QCM-D can be an important tool to study the binding of NLP formulations or other nanoparticles with cell membranes under dynamic flow, which very often differs from nanoparticle uptake under static conditions.

## Abstract 2

The investigation of the effect of the protein corona on the cellular uptake of nanoliposome formulations in real-time under flow conditions by quartz crystal microbalance with dissipation as a biomimetic *in vitro* technique.

## Graphical abstract

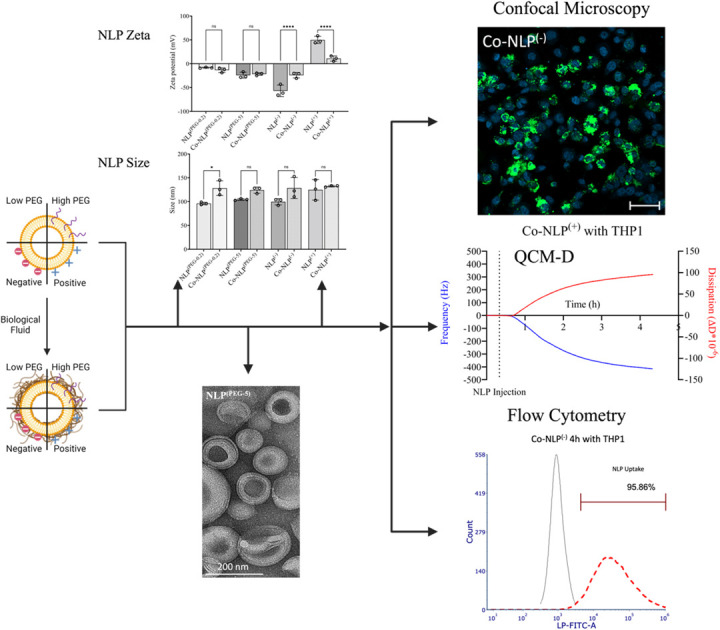

## 1 Introduction

With the ability to prolong drug circulation times and reduce systemic toxicity, nanoparticles offer a powerful approach for detecting or treating disease through a variety of delivery routes.^1–3^ Despite the growing interest in nanoparticle delivery systems, the clinical translation of nanoparticles is still limited.^4^ This is due in part to a limited understanding of the interactions of nanoparticles with their local biological environment and their cellular uptake.^5^ When a nanoparticle is immersed in a biological fluid, such as blood or saliva, a complex coating of proteins and other biomolecules forms on the nanoparticle, called the protein corona.^1,4–8^ Once the protein corona forms, the synthetic identity of the nanoparticle is altered, conferring a new biological identity.^1,9^ This results in differences in the biodistribution, half-life, and efficacy of nanoparticles in the body, creating a gap between the *in vitro* and *in vivo* performance of these drug delivery systems.^4,5,7,10^ The nature of the protein corona has been reported to vary based on the physicochemical properties of the nanoparticle and the environmental factors surrounding the nanoparticle in biological fluids.^11^ For instance, Xiao *et al.* reported that varying the surface charge of polystyrene nanoparticles altered the composition of the protein corona and affected macrophage polarization, while variations in particle size affected the abundance of the protein corona, but did not change the nature of the proteins absorbed on the surface.^12^

Among the many types of nanoparticles, nanoliposomes (NLPs) are a versatile drug delivery platform of great interest in nanomedicine and are an industry standard, involved in the majority of FDA-approved nanomedicines.^3,6,7^ They are composed of phospholipid bilayers with an aqueous core, which allows them to carry both hydrophilic and hydrophobic drugs, enabling the delivery of a wide variety of therapeutics.^3,13,14^ One of the first NLPs was a liposomal doxorubicin formulation (Doxil®) approved by the FDA in 1995.^3^ Since then, several other NLP formulations have been approved to reduce the side effects of chemotherapeutic agents such as paclitaxel.^3,15^ An extensive library of available phospholipids can endow liposomes with a variety of physicochemical properties and surface functionalities. With variations in the protein profiles of the protein corona formed on different liposome formulations, it is suggested that the composition of the protein corona is influenced by surface charge and chemistry.^16^ Variations in the protein corona composition were also observed when the NLPs were incubated in either mouse or human plasma, indicating that the protein source alters the protein corona formation and that success in using animal models does not directly translate to their clinical success.^16^ Interestingly, protein coronas formed on NLPs in hypercholesterolemic mice were enriched in apolipoproteins and depleted of most complement proteins, except C9. This resulted in an enhanced inflammatory response and altered biodistribution of the NLPs when compared to healthy mice. The authors then suggested that the metabolomic profile can be used as a new way to personalize nanomedicine treatment.^17^ Similarly, conjugated NLPs with albumin-binding domains enriched the protein corona with albumin by a factor of eight, resulting in a longer blood circulation time, more significant accumulation at tumor sites, and higher antitumor efficacy in a mouse model.^18^

Another consideration when investigating the effect of the protein corona on the fate of nanoparticles *in vivo* is the effect of the flow rate on their uptake. When administered systemically, nanoparticles enter the bloodstream and circulate throughout the body under a dynamic flow. Many *in vitro* studies do not take the dynamic nature of blood flow into account in their experiments.^19^ For example, when gold nanoparticles were incubated with cells, nanoparticle sedimentation led to increased uptake by cells and confounded the data; this is unlikely to happen under dynamic flow.^20^ New *in vitro* methods for thorough and accurate assessment of the effects of the protein corona on nanoparticle performance under dynamic flow are therefore needed to enable us to better correlate *in vitro* data with *in vivo* models and thus improve clinical translation.^21^ This has motivated several authors to develop microfluidic devices to simulate blood flow conditions in order to obtain more biomimetic results.^19,22^ Quartz crystal microbalance with dissipation (QCM-D) is also a promising *in vitro* technique capable of mimicking physiological blood flow conditions and studying the effect of the protein corona on the nanoparticles' fate in real-time. In this technique, shifts in the resonant frequency of a piezoelectric crystal, recorded as frequency changes (Δ*f*), are used to track mass changes occurring at the surface of the sensor, while the dissipation (Δ*D*) of the crystal's oscillation is simultaneously registered to track changes in the viscoelastic properties of the surface.^23^

Due to this ability to monitor both the mass and viscoelastic properties of a surface under flow conditions in real-time and *in situ*, QCM-D has been widely used to investigate protein interactions with various analytes and protein adsorption on different surfaces.^23–26^ Some examples include its use to study the adsorption of bovine serum albumin on different graphene surfaces,^24^ to investigate the interaction of plasma proteins and adhesion to multiple surfaces,^26^ to study the nanoparticle interactions with various biomolecules,^23,27,28^ and to demonstrate the impact of aluminum nanoparticles on human platelet function.^27^ QCM-D has also become increasingly popular to study cell–cell adhesion, binding kinetics, and characterization of cell cytoskeletal mechanics.^29,30^ An example is the use of QCM-D to investigate the interaction of glycans present in tumour cells and their metastases with lectins.^31^

The aim of this study was to introduce QCM-D as a relevant technique to investigate the effect of the protein corona on the cellular interaction of various NLP formulations under dynamic flow mimicking blood flow. We stipulated that the composition of the protein corona would be affected by the physicochemical properties of the nanoparticles, resulting in a different cellular uptake profile, depending on the cell type, and that these changes could be quantified in real-time using QCM-D. For this proof-of-concept study, two PEGylated NLPs with either a low or a high PEG ratio, as well as negatively and positively charged NLPs, were synthesized. Their charge and size were assessed before and after the formation of a protein corona using dynamic light scattering (DLS), nanoparticle tracking analysis (NTA) and transmission electron microscopy (TEM). The subsequent interactions of bare and corona NLPs with macrophage and human melanoma cell lines were determined using QCM-D. The human macrophage THP1 cell line was chosen as a model due to its role as a primary immune cell involved in the phagocytosis of nanoparticles.^32^ The human malignant melanoma A375 cell line was selected as the cancer cell model, since late-stage melanoma typically responds poorly to treatment and is highly metastatic. The effect of the protein corona on cellular uptake was further studied by confocal microscopy and flow cytometry to corroborate the QCM-D results.^33–36^

## 2 Materials and methods

### 2.1 Materials

1,2-distearoyl-*sn*-glycero-3-phosphocholine (DSPC) and 1,2-distearoyl-*sn*-glycero-3-phosphoethanolamine-*N*-[carboxy(polyethylene glycol)-2000] (DSPE-PEG2000) were purchased from Avanti Polar Lipids (Alabaster, AL, USA). Positively charged lipids 1,2-dioleoyl-3-trimethylammonium propane (DOTAP) and negatively charged lipids 1,2-dioleoyl-*sn*-glycero-3-phosphoserine (DOPS) were purchased from Cayman Chemical Company (Ann Arbor, MI, USA). Cholesterol (Chol) and phorbol 12-myristate 13-acetate (PMA) were acquired from Sigma-Aldrich (St. Louis, MO, USA). The human melanoma cell line (A375) was generously donated by Dr Ian Watson. The human monocyte cell line (THP1) was purchased from ATCC (Manassas, VA, USA). *N*-(Fluorescein-5-thiocarbamoyl)-1,2-dihexadecanoyl-*sn*-glycero-3-phosphoethanolamine (FDHPE), Dulbecco's Modified Eagle Medium (DMEM), Roswell Park Memorial Institute (RPMI) 1640 Medium, heat-inactivated fetal bovine serum (FBS), penicillin-streptomycin (P/S), 0.25% trypsin, 2-mercaptoethanol, 0.01% poly-l-lysine solution (PLL), 0.4% Trypan blue, Hoechst 33342, EBioscience™ Fixable Viability Dye eFluor™ 780, and Ebioscience™ Flow Cytometry Staining Buffer were purchased from Thermo Fisher Scientific (Waltham, MA, USA). Human pooled plasma NA EDTA was purchased from Innovative Research Inc. (Novi MI, USA). Silicon dioxide (QSX 303) crystals were purchased from Nanoscience Instruments (Phoenix, AZ, USA). 37% paraformaldehyde (PFA) was purchased from Bio Basic (Markham, ON, Canada), and 100% ethanol from Commercial Alcohols (Boucherville, QC, Canada).

### 2.2 Synthesis of nanoliposomes

**Fig. 1:**
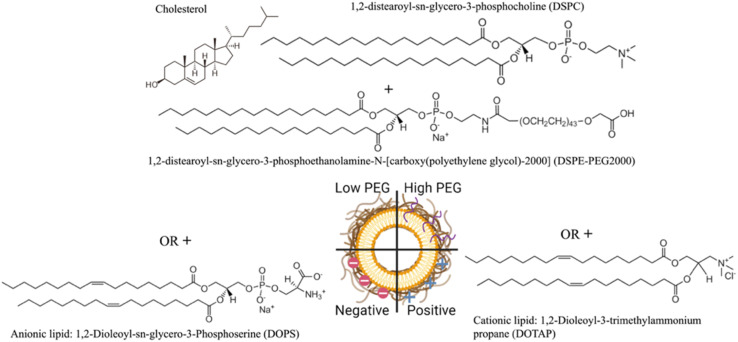
Illustration of nanoliposome formulations investigated in this study.

The NLP formulations consisted of two PEGylated NLPs composed of DSPC/Chol/DSPE-PEG2000 with a 65/34.8/0.2 or 65/30/5 molar ratio, negatively charged NLPs consisting of DSPC/Chol/DOPS with a 50/30/20 molar ratio, and positively charged NLPs composed of DSPC/Chol/DOTAP with a 50/30/20 molar ratio prepared by the ethanol injection method (Fig. 1).

Briefly, for the PEGylated NLPs, DSPE-PEG2000 in chloroform as received was dried in a rotary evaporator, resuspended in 100% ethanol, and stored at −20 °C until use. This DSPE-PEG2000 in ethanol and phospholipids (DSPC) were then dissolved in 100% ethanol to a total volume of 5 ml and injected at a constant rate into a stable vortex formed with 45 ml of ultrapure water. This 50 ml solution was stirred at a low speed for 15 minutes and then concentrated using a rotary evaporator until a volume of 9 ml. 1 ml of 10x phosphate-buffered saline (PBS from Sigma Aldrich Product #P4474, pH 7.2) was added to the solution and then stored at 4 °C. Fluorescent versions of the liposomes were made by reducing DSPC by 1% mol and then incorporating FDHPE at 1% mol in all formulations. The fluorescent liposomes were prepared by the same method described above.

### 2.3 Size and charge measurements

The NLP formulations were diluted 1 : 50 000 in PBS or 1 : 10 in ultrapure water for NTA (NTA-Nanosight NS300, Malvern, UK) and DLS (Brookhaven Zeta-PALS light scattering analyzer, New York, NY, USA), respectively, to record their concentration, mean size, and zeta potential at room temperature (*n* = 6 : 3 times from 2 different preparations) immediately after the sample fabrication.

### 2.4 Transmission electron microscopy

NLPs were drop-cast onto a carbon-copper grid and stained with uranyl acetate for contrast. The transmission electron microscopy (TEM) images were acquired using a Thermo Scientific Talos F200X G2 (S)TEM.

### 2.5 Protein corona formation on the nanoliposomes

NLP solutions were suspended in pooled human plasma Na EDTA at a ratio of 1 : 1 (v/v) for two hours at room temperature on a low-speed rotary mixer. Nanoliposome–protein complexes were then isolated by ultracentrifugation at 120 000 RCF for 15 minutes and resuspended in 1x PBS, repeated three times to remove any loosely bound proteins.

### 2.6 Cell culture

A375 and THP1 cells were cultured in DMEM and in RPMI respectively under a 5% CO_2_ atmosphere at 37 °C in a fully humidified incubator. Both media were supplemented with 10% FBS and 1% P/S; for THP1 cells, the media also contained 50 μM 2-mercaptoethanol. To differentiate the THP1 cells to the M0 phenotype, cells were harvested at confluence and spun down at 200 RCF for 5 minutes. The THP1 cells were then resuspended in 5 ml of RPMI, and 1 million cells per well were seeded in a 6-well plate supplemented with PMA at 10 ng ml^−1^. After 24 hours of incubation, the medium containing PMA was aspirated and replaced with 3 ml of fresh RPMI, and the cells were incubated for another 24 hours. Cells were then harvested using 0.25% trypsin, and their viability was determined *via* 0.4% Trypan blue staining using a Countess II Cell Counter (Thermo Fisher, Waltham, MA, USA).

### 2.7 QCM-D experiments

Silicon dioxide (QSX 303) crystals were cleaned according to the manufacturer's protocol. Briefly, the sensors were plasma-gas treated with a PE-50 (Plasma Etch, Carson City, NV, USA) for 10 min, immersed in 2% sodium dodecyl sulfate (SDS) for 30 min at room temperature, rinsed thoroughly in ultrapure water, dried with nitrogen gas, and plasma-gas treated again for 10 min. To regenerate the sensors after use, 0.25% trypsin was employed to detach any remaining cells. The sensors were thoroughly rinsed in ultrapure water and dried with nitrogen gas before applying the cleaning protocol. The sensors were then soaked in 180 μl of 0.01% PLL solution for 12 hours, then rinsed in ultrapure water, dried with nitrogen gas, and exposed to UV light for 20 minutes to sterilize them prior to their use with cells. Harvested A375 cell suspensions were counted and seeded in 12-well plates at 150 000 cells per sensor suspended in 2 ml DMEM. Cell-coated sensors were incubated for 36 hours before experiments to allow cells to attach to the sensor surface. For THP1, the cells were differentiated directly on the sensors in the 6-well plates. All QCM-D measurements were performed with a Q-Sense E4 unit (Biolin Scientific, Gothenburg, Sweden). Prior to each measurement, the temperature of the flow modules and the degassed media was equilibrated to 37 °C. The cell-coated sensors were then rinsed in PBS to remove unbound cells. The quality, density and uniformity of the cell monolayer were verified by light microscopy. The sensors were mounted in the QCM-D flow modules into which serum-free DMEM or RPMI was flowed at 10 μl min^−1^. Under this constant flow, the chambers were allowed to equilibrate for one hour to establish the baseline, and this condition was maintained for 20 minutes to ensure baseline stability. Subsequently, bare or protein corona NLP formulations, diluted in a 1 : 10 ratio in serum-free DMEM or RPMI, were flowed through the chambers at 10 μl min^−1^ for 4 hours to allow the nanoliposomes to be taken up by the cells. The cell sensors were then carefully removed from the flow modules and observed under a light microscope to ensure that the cell monolayer was not significantly altered by the process. After each experiment, the temperature controller was turned off and the flow modules and tubing were cleaned with a sequence of ultrapure water, 2% Hellmanex solution, ultrapure water, 2% SDS, ultrapure water, 70% ethanol, and finally ultrapure water, for 10 min each, at a flow rate of 250 μl min^−1^. Upon the completion of the cleaning protocol, the flow modules were dried with nitrogen gas. All QCM-D data are presented from the third overtone, as its penetration depth coincides with the basal region of the cell monolayer.^30^

### 2.8 Fluorescent microscopy analysis

A375 and THP1 cells were seeded at a density of 20 000 cells per well in 96-well glass bottom plates, pre-coated with 40 μl of 2% sterile gelatin. 200 μl of DMEM, or RPMI containing PMA at 10 ng ml^−1^, was then added, and the cells were allowed to attach for 24 hours. For the THP1 cells, the medium was changed to RPMI without PMA and the cells were incubated for an additional 24 hours to differentiate to M0. Each well was then washed 3 times with PBS. 20 μl of fluorescent bare or 100 μl of fluorescent corona NLPs were mixed with 180 μl of serum-free DMEM or RPMI and incubated with the cells for 1 or 4 hours. The supernatant was removed, and the wells were washed three times with PBS and fixed with 100 μl of 4% paraformaldehyde in PBS for 10 minutes. The paraformaldehyde was removed from the wells and washed three times with PBS. Finally, 100 μl of Hoechst solution diluted 1 : 1000 in PBS was added to each well. Imaging was performed with a Zeiss LSM800 confocal microscope at x40 using a constant laser intensity and gain throughout the imaging process for all samples.

### 2.9 Flow cytometry experiments

A375 and THP1 cells were seeded at a density of 1 × 10^6^ cells per well in a 6-well plate. A375 cells were allowed to adhere to the plate for 24 hours, and THP1 cells were differentiated to M0 as described above. The medium was then removed, and each well was washed 3 times with PBS. 50 μl of bare fluorescent nanoliposomes and 250 μl of corona-containing fluorescent NLPs were mixed with 1950 μl or with 1750 μl of serum-free DMEM or RPMI, to label A375 and THP1 cells, respectively; NLP solutions were then added to the wells and incubated for 1 or 4 hours. A higher concentration of the protein corona NLPs was used to account for the loss of particles occurring during the isolation of the protein–NLP complex by centrifugation. The NLP solutions were removed from each well and washed 3 times with PBS. The cells were detached from the surface using 0.5 ml of 0.25% trypsin for 5 minutes, collected using either 1 ml of DMEM or RPMI, centrifuged at 300 RCF for A375 cells and 200 RCF for THP1 cells for 5 minutes and resuspended in 1 ml of PBS twice. 1 μl of EBioscience™ Fixable Viability Dye eFluorTM 780 was added to the solutions and further incubated for 30 minutes, centrifuged and resuspended in PBS twice before being resuspended in 2% paraformaldehyde to fix the cells for 15 minutes. Finally, the suspension was centrifuged and washed in PBS before resuspending the fixed cells in EBioscience™ Flow Cytometry Staining Buffer and stored at 4 °C overnight. NLP uptake was then quantified using an Attune™ CytPix™ flow cytometer (Thermo Fisher Scientific, Waltham, MA, USA).

### 2.10 Determination of protein corona composition *via* mass spectrometry

Each NLP–protein complex was loaded onto a single stacking gel band to remove lipids, detergents, and salts. The gel band was reduced with DTT, alkylated with iodoacetic acid, and digested with trypsin. Extracted peptides were resolubilized in 0.1% formic acid and loaded onto a Thermo Acclaim Pepmap (Thermo, 75 μm ID × 2 cm C18 3 μm beads) precolumn and then onto an Acclaim Pepmap Easyspray (Thermo, 75 μm × 15 cm with 2 μm C18 beads) analytical column using a Dionex Ultimate 3000 μHPLC at 250 nl min^−1^ with a gradient of 2–35% organic (0.1% formic acid in acetonitrile) over 3 hours. Peptides were analyzed using a Thermo Orbitrap Fusion mass spectrometer operating at 120 000 resolution (FWHM in MS1) with HCD sequencing (15 000 resolution) at top speed for all peptides with a charge of 2+ or greater. The raw data were converted into *.mgf format (Mascot generic format) for searching using the Mascot 2.6.2 search engine (Matrix Science) against human protein sequences (Uniprot 2023). The database search results were loaded onto Scaffold Q + Scaffold 5.0.1 (Proteome Sciences) for statistical processing and data visualization. The plasma control spectrum, plasma treated in the same way as the NLPs, was subtracted from the total spectrum count of each protein in each particle group to determine the extent to which each protein was enriched or depleted compared to the control group.

### 2.11 Statistical analysis

All quantitative experiments were carried out independently in biological triplicates (*n* ≥ 3) unless indicated otherwise. An unpaired Welsch's *t*-test and Welch one-way ANOVA were used to assess the statistical significance between groups at 95% confidence. The data were considered significant when *p* < 0.05 (* < 0.05, ** < 0.01, *** < 0.005, and **** < 0.0001). All statistics were performed using Prism GraphPad 10 software.

## 3 Results

### 3.1 The protein corona alters nanoliposome surface properties

**Fig. 2:**
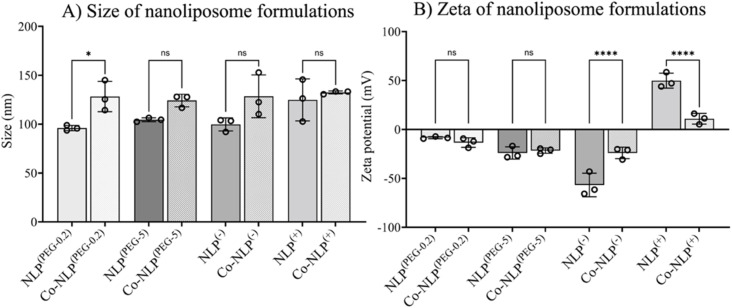
(A) Size distribution of NLPs as measured by NTA and (B) zeta potential of NLP formulations before and after protein corona formation, showing a lower effect of the protein corona on particle size, but inducing more significant variation in the zeta potential of the NLPs.

Table 1 summarizes the NTA and zeta potential measurements for NLP groups along with the abbreviations used for sample identification when reporting the results. The ethanol injection method yielded relatively monodispersed NLPs with concentrations of approximately 6 × 10^12^ particles per ml for all groups (Fig. 2A). Prior to protein corona formation, the NTA indicated an average NLP size of approximately 100 nm with an increasing trend in their size after protein corona formation, suggesting that a protein layer was formed around the particles. However, the increase in size was only significant for Co-NLP^(PEG-0.2)^ compared to NLP^(PEG-0.2)^ (99 ± 3 nm *vs.* 128 ± 16 nm). Co-NLP^(+)^ had the smallest change in particle mean size, likely because the proteins are more densely packed, due to strong electrostatic interactions between these particles and the plasma proteins, which are mostly negatively charged.^4^ Size increases of this magnitude are consistent with the literature, which reports similar increases in the PEGylated, negatively, and positively charged NLPs after protein corona formation.^37–40^

**Table 1:** Abbreviations and characteristics of NLP formulations used in this study

Nanoliposome (NLP) formulations	Ratio (mol%)	Abbreviation		Size (nm)		Zeta (mV)	
		Bare	Coronated	Bare	Coronated	Bare	Coronated
DSPC/Chol/DSPE-PEG2000	65/34.8/0.2	NLP^(PEG-0.2)^	Co-NLP^(PEG-0.2)^	99 ± 3	128 ± 16	−8 ± 1	+13 ± 5
DSPC/Chol/DSPE-PEG2000	65/30/5	NLP^(PEG-5)^	Co-NLP^(PEG-5)^	104 ± 2	124 ± 6	−24 ± 6	−22 ± 3
DSPC/Chol/DOPS	50/30/20	NLP^(−)^	Co-NLP^(−)^	100 ± 7	129 ± 22	−57 ± 12	−24 ± 6
DSPC/Chol/DOTAP	50/30/20	NLP^(+)^	Co-NLP^(+)^	125 ± 21	132 ± 2	+50 ± 8	+11 ± 5

**Fig. 3:**
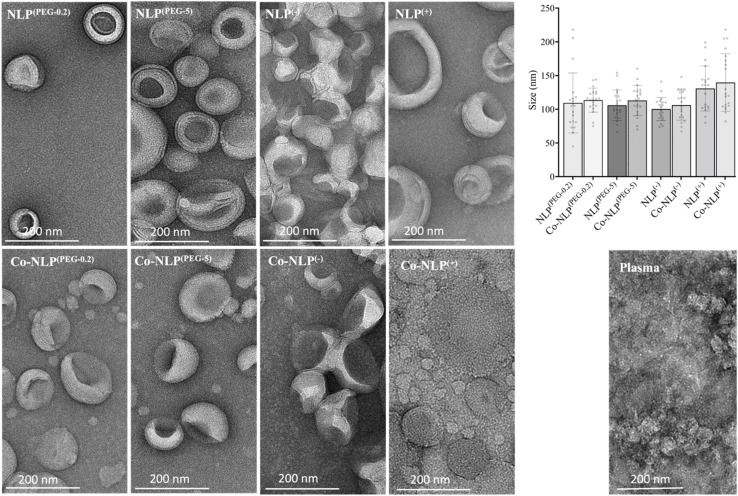
Morphology of the NLP formulations before and after the protein corona formation, as revealed by TEM. Each image depicted a lighter external ring enclosing a dark center, consistent with the nanoliposome's spherical shape and aqueous core with a visible bilayer, as reported in the literature. The size of NLP formulations is between 100 nm and 140 nm, which corroborates with the sizes obtained by the NTA measurements. Scale bar is 200 nm.

As expected, changes in zeta potential were observed for protein corona NLPs (Fig. 2B). The mean charge was – 8 ± 1 mV for NLP^(PEG-0.2)^, −24 ± 6 mV for NLP^(PEG-5)^, −57 ± 12 mV for NLP^(−)^ and +50 ± 8 mV for NLP^(+)^, with an order of stability in dilute buffer solution of NLP^(−)^ > NLP^(+)^ > NLP^(PEG-5)^ > NLP^(PEG-0.2)^, indicating that the NLPs are colloidally stable and are not prone to aggregation. The exposure of NLP formulations to the plasma proteins alters the zeta values for all samples, but more significantly for the charged NLPs, *i.e.*, Co-NLPs^(−)^ and Co-NLPs^(+)^ (−57 v/s −24 and + 50 v/s +11, respectively). The TEM image analysis indicated that the size and morphology of NLPs were maintained for most of the formulations with an increasing trend in particle size after protein corona formation (Fig. 3). Some NLP aggregation was observed for NLP^(PEG-0.2)^, revealing its colloidal stability (Fig. S1†), which was also revealed by zeta potential analyses. The presence of small nanoparticles surrounding Co-NLPs^(+)^ is most likely associated with the different types of proteins in plasma, in particular with the four main endogenous lipid particles, whose nature generally varies according to the physicochemical properties of the NLPs, such as size and zeta potential.^41,42^

### 3.2 QCM-D shows that cell-NLP attachment is affected by the protein corona

**Fig. 4:**
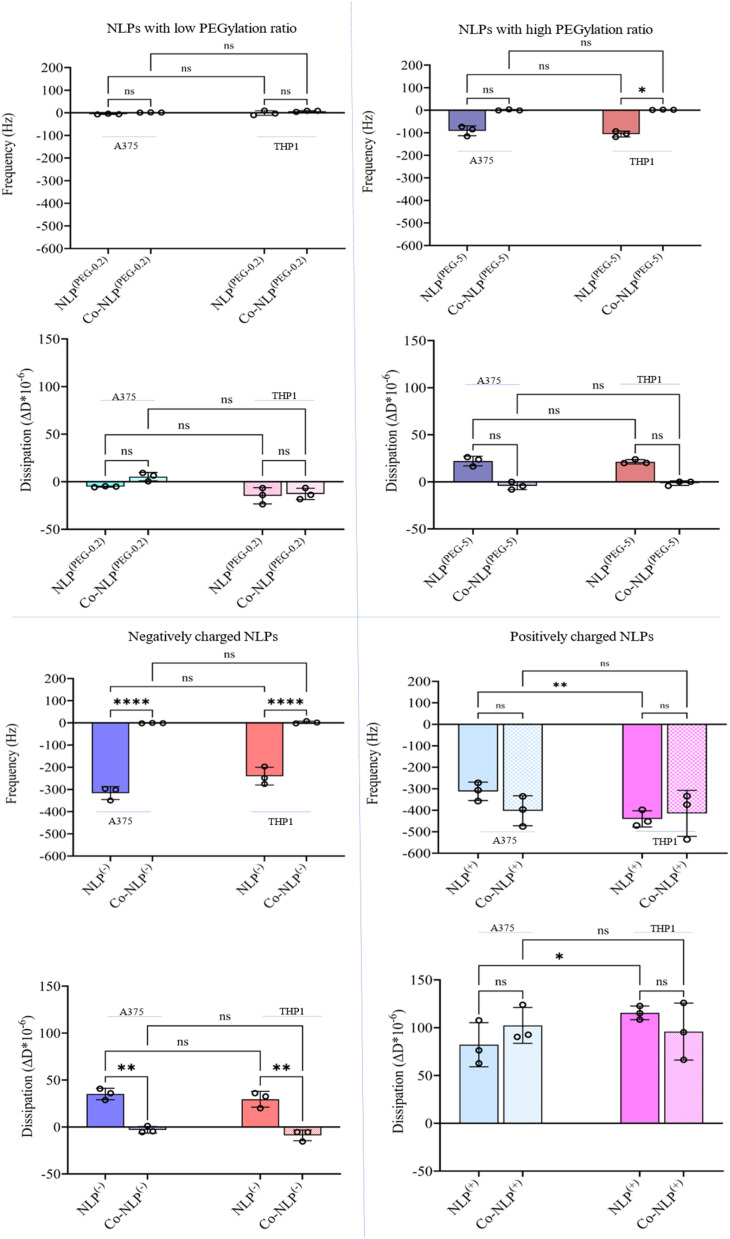
Δ*f*_3_ and Δ*D* shifts after flowing the bare and corona NLPs over A375 and THP1 cells indicating that charge, protein corona, and cell type have significant effects on cellular uptake.

Fig. 4 compiles the frequency (Δ*f*_3_) and dissipation (Δ*D*) shifts as a result of mass increase on the QCM-D quartz crystal following the exposure of the cell monolayers (Fig. S2†) to NLPs, monitored for 4 hours (Fig. S3†). The shifts were different for each NLP formulation.

Flowing NLP^(PEG-0.2)^ over the A375 cells (Fig. S4-A†) and the THP1 cells (Fig. S4-B†) led to a frequency shift of 6 Hz and 1.5 Hz with a dissipation shift of 5 Δ*D* and 15 Δ*D*, respectively. A decrease in frequency (blue lines) indicates an increase in mass on the crystal, while an increase in dissipation (red lines) indicates an increase in viscoelasticity at the surface, and *vice versa*. The frequency and dissipation changes were not significant for NLP^(PEG-0.2)^ flowing over the A375 and THP1 cells after protein corona formation (A375 cells: Δ*f*_3_ = 1 Hz with a Δ*D* = 5; THP1 cells: Δ*f*_3_ = 7 Hz with a Δ*D* = 1).

Both NLP^(PEG-0.2)^ and Co-NLP^(PEG-0.2)^ produced a frequency shift of less than 1 Hz h^−1^ and a dissipation drift of less than 0.15 Δ*D*/h (a drift that is expected for a pristine crystal surface at room temperature when water flows through the chamber^43^). This implies that only very few NLPs were attached to the surface of either cell line. However, a greater cellular attachment was observed after flowing NLP^(PEG-5)^, as evidenced by a larger frequency and dissipation shifts (A375 cells: Δ*f*_3_ = 91 Hz and Δ*D* = 22; THP1 cells: Δ*f*_3_ = 106 Hz and Δ*D* = 21). The difference in cellular uptake disappeared for the Co-NLP^(PEG-5)^ group. In contrast, both positively and negatively charged NLPs, bare or coronated, showed a higher cellular attachment than the PEGylated NLPs. Flowing NLPs^(−)^ caused frequency and dissipation shifts for both cell types much higher than those of the PEGylated NLPs with a higher shift for A375 cells (Δ*f*_3_ = 316 Hz and Δ*D* = 35) compared to the THP1 cells (Δ*f*_3_ = 240 Hz and Δ*D* = 29). These values were significantly reduced (to the same level as NLPs^(PEG-0.2)^) for Co-NLPs^(−)^ for both cell types, with no significant difference between them, indicating that the protein corona has drastically affected the cellular attachment of NLPs^(−)^. Flowing NLPs^(+)^ over the A375 or THP1 cells resulted in a frequency shift of 312 Hz with a dissipation shift of 82, and a frequency shift of 440 Hz with a dissipation shift of 115, respectively. Even with the protein corona, NLPs^(+)^ maintained their effect, changing the frequency and dissipation at the same level as the bare NLPs^(+)^ regardless of the cell lines over which they were flowed (Δ*f*_3_ = 403 Hz and Δ*D* = 102 for A375 cells; Δ*f*_3_ = 415 Hz and Δ*D* = 96 for the THP1).

### 3.3 Confocal microscopy analyses on NLP formulations' cellular uptake corroborate the QCM-D results

**Fig. 5:**
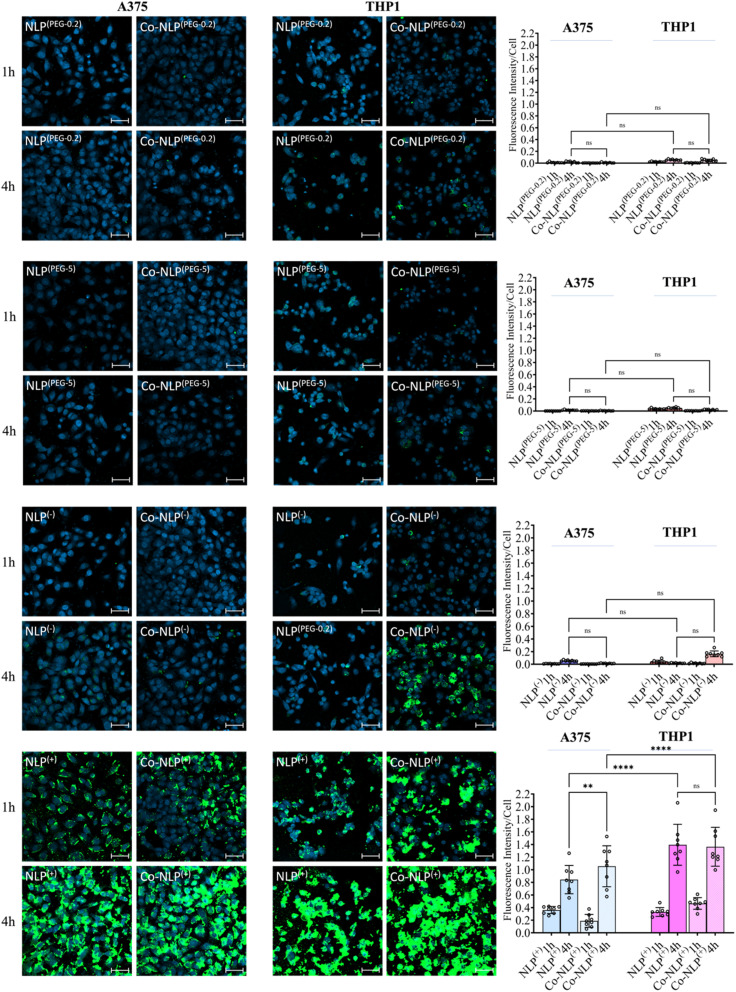
Confocal microscopy images of the uptake of bare and coronated NLPs by A375 and THP1 cells after 1 and 4 hours. The scale bar is 50 μm. Very low NLP uptake of PEGylated and negatively charged NLPs before and after the protein corona formation, but high uptake of positively charged NLPs by A375 cells was observed. The NLP uptake tended to be higher in THP1 macrophages when compared to A375 cells.

DAPI staining of nuclei and FITC staining of the NLPs after 1 hour and 4 hours of incubation with each cell line are shown in Fig. 5, S5-a and S5-b.† Both the bare and protein corona low or high PEGylation NLPs incubated with the A375 cells showed very low fluorescence intensity in the FITC channel, indicating very low cellular uptake of these NLPs. Although slightly higher amounts of the PEGylated NLPs were detected in the THP1 cells, their cellular uptake remains very low. A significant increase in cellular uptake was observed when NLPs^(−)^ and Co-NLPs^(−)^ were incubated with A375 cells or THP1 cells. The cellular uptake was the highest for both NLPs^(+)^ and Co-NLPs^(+)^ in both cell types. Overall, the confocal microscopy results were in accordance with the data obtained with QCM-D for the cellular uptake of the nanoliposome formulations. However, the higher cellular uptake of NLPs observed in confocal microscopy images, particularly for THPI cells, can be explained by the difference in the assessment of the cellular uptake under dynamic (QCM-D) and static flow conditions (confocal microscopy).

### 3.4 Quantification of NLPs' cellular uptake by flow cytometry supports the QCM-D data

**Fig. 6:**
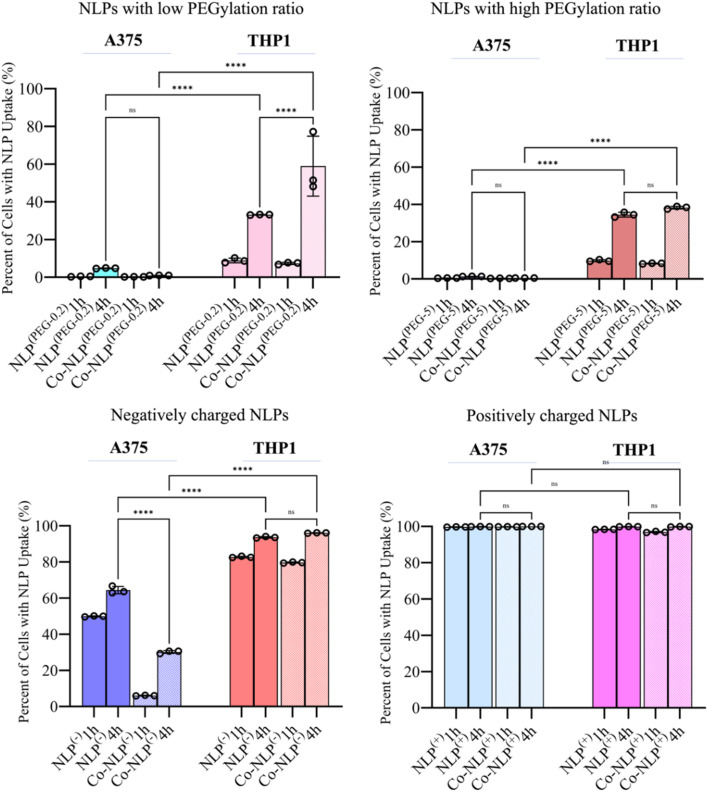
Flow cytometry data for the uptake of NLP after 1 hour or 4 hours of incubation with A375 or THP1 cells. Overall, the results indicated an increase in cellar uptake after four hours of incubation. Significantly higher cellular uptake was observed in THP1 compared to A375 cells for all groups with almost 100% uptake for positively charged NLPs on both cell types.

Fig. 6 shows the percentage of the cell population that is positive for each fluorescently labeled bare or corona NLP at 1 and 4 hour time points, obtained *via* analyzing the corresponding histograms and gating strategy (Fig. S6 and S7†). The flow cytometry results were similar to the confocal microscopy observations. Regardless of the time point, and despite their higher attachment to the cell surface as observed in QCM-D results, less than 5% of A375 cells were FITC positive when they were incubated with PEGylated NLPs. This may suggest that these NLPs were loosely attached to the cell surface and washed out by the running flow. A significant increase in the number of FITC-positive THP1 cells (34% and 38% for Co-NLPs^(PEG-5)^ and Co-NLPs^(PEG-5)^, respectively) was however observed when the cells were exposed to these NLPs.

Nevertheless, the number of FITC positive THP1 cells was higher with a significant increase at the 4 hour time point (<10% for the PEGylated bare NLPs after 1 hour v/s ∼40% at 4 hours). The cell uptake for PEGylated NLPs with the protein corona increased again significantly at the 4 hour time point in THP1 cells (58% for NLPs^(PEG-0.2)^ and 33% for NLPs^(PEG-5)^). Even after 4 hours, the FITC-positive A375 cells were still less than 2% for these NLP formulations, despite their higher attachment to the cell surface as observed in the QCM-D results. For the negatively and positively charged NLPs, there were more FITC-positive cells in both cell types. The uptake of NLPs^(−)^ by A375 cells was significantly lower compared to their uptake by THP1 cells after 4 h (64% *vs.* 96%). This number decreased by 30% in A375 cells for Co-NLPs^(−)^, whereas the number of FITC-positive THP1 cells remained above 96%, with no significant difference between 1 and 4 hours of incubation. For NLPs^(+)^, regardless of whether they were bare, protein-coronated or incubated for any length of time, the number of FITC-positive cells was almost 100%, consistent with their favorable attachment to cells, as was reflected in high Δ*f*_3_ and Δ*D* values in QCM-D data.

### 3.5 Protein corona profiles vary according to nanoliposome formulations

**Fig. 7:**
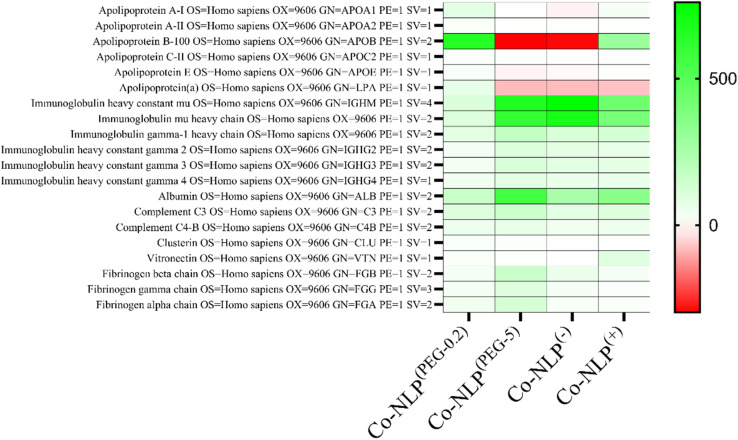
Heat map of the major proteins on NLPs after the formation of the protein corona. The color coded bars indicate an enriched (green) or a decreased protein content in the sample compared to the plasma control group. An increase of immune-globulins and a decrease of apolipoprotein levels were observed for protein-coronated charged NLPs.

Fig. 7 displays a heat map of several proteins, which are commonly reported in the protein corona of liposomes.^7,44,45^ In this map, green, white and red colors represent an enriched protein content, a similar protein content, and a decreased protein content, respectively, when compared to the control group, *i.e.*, plasma centrifuged in the same manner as the NLPs; the deeper the color, the greater the difference in protein content.

Overall, the protein composition between the liposome groups was comparable, with all having similar levels of complement proteins, immunoglobulins, and various other proteins (Fig. S8-a and S8-b†). However, Co-NLPs^(−)^ and Co-NLPs^(PEG-5)^ had increased amounts of immunoglobulin M (IgM) fragments (immunoglobulin heavy constant mu and immunoglobulin mu heavy chain), while having decreased amounts of several apolipoproteins. Co-NLPs^(PEG-5)^ also had increased amounts of albumin and fibrinogen chains, Co-NLPs^(PEG-0.2)^ had increased levels of apolipoproteins, and Co-NLPs^(+)^ had increased levels of vitronectin.

## 4 Discussion

QCM-D is commonly used to investigate proteins/cells and nanoparticle interactions, as well as cellular mechanics.^23–31,46^ However, there are no reports of using QCM-D to investigate the effect of the protein corona on nanoparticle interactions with and attachment to cells. The aim of this study was to introduce QCM-D as a viable tool to elucidate the effect of the protein corona on NLP binding to cells as the first step in the cellular uptake pathway. Four NLPs, differing in chemical composition and charge, were prepared and exposed to cell monolayers after the formation of the protein corona on the nanoparticles in a QCM-D setup. Frequency and dissipation shifts, resulting from NLP attachment to cells, were monitored under dynamic flow and compared to bare NLPs. Fluorescence microscopy and flow cytometry were used to corroborate the QCM-D data with the subsequent cellular uptake of NLPs.^33–35^

Prior to the QCM-D analyses, the formation of a protein corona on NLPs was confirmed by size, zeta potential and TEM analyses. Particularly, the presence of very small nanoparticles around Co-NLPs and their absence in bare NLPs in TEM images suggested the formation of a protein corona around NLPs. The formation of the protein corona on nanoparticles upon exposure to biological fluids has been widely reported in the literature.^47^ This protein corona alters the physicochemical properties of the NLPs, such as size and zeta potential, creating a new biological identity of the NLPs.^7^ In human plasma itself, there are four main endogenous lipid nanoparticles, namely chylomicrons (75–1200 nm), high-density lipoproteins (8–12 nm), low-density lipoproteins (19–25 nm) and very low-density lipoproteins (VLDL) (30–80 nm), which are responsible for the transport of lipids through the blood.^41,42^ The presence of small particles between 30 and 80 nm in the TEM images of protein-coronated PEGylated and positively charged NLPs suggests the association of VLDL particles with them. VLDL particles are reported to have a zeta potential of −15.1 mV, so they are repelled by the negatively charged NLPs, which explains their lower abundance in the TEM images of negatively charged NLPs.^42^

When proteins surround a nanoparticle, their diameter can increase by a few to several tens of nanometers. If the osmotic pressure of the protein layer is high enough, it can also cause a decrease in particle size.^38,44^ When a large increase in nanoparticle size is observed, it is attributed to particle aggregation as a result of protein absorption to the surface.^44,48^ Our NLPs tend to become larger after the protein corona formation on their surface, but not significantly, indicating that the protein corona is not very thick, the proteins do not cause NLP aggregation, and they are not massively aggregated around the NLPs. In addition to size, the protein corona tends to normalize the charge of the nanoparticles. Since most proteins in plasma are negatively charged, coronated nanoparticles often tend toward −20 mV (approximately the charge of plasma).^4,38,44^ All NLP groups showed a trend in zeta potential toward −20 mV, confirming the presence of a protein corona on their surface. These results are consistent with the literature, which reports similar changes in nanoparticles when exposed to plasma.^39,40,49^

The QCM-D data indicated that charge, colloidal stability, and the presence of functional groups, such as carboxylic acids, are parameters that influence the cellular attachment. As depicted in Fig. 3, prior to the formation of the protein corona on NLPs, NLP^(+)^ showed superior interactions with the negatively charged cell membrane. The low PEGylated NLPs had the lowest cellular uptake, probably due to a low surface charge leading to little interaction with the negatively charged cell membrane^50–52^ and lower colloidal stability (low zeta value) that could cause aggregation of these NLPs (Fig. S1†).^53,54^ A higher degree of PEGylation increased the zeta potential, thereby increasing the colloidal stability. Since the PEG used here had a carboxylic acid group, it resulted in improved electrostatic interactions between highly PEGylated NLPs and the few positively charged domains on the cell surface compared to the low PEGylated NLPs.^51,52^ Although negatively charged, the higher colloidal stability of NLP^(−)^ seemed to play an important role in the binding of these NLPs to positively charged domains of the cell membrane compared to the PEGylated NLPs.^50–52,55^ As expected, the protein corona surrounding the NLP affected their interaction with cells.^56^ The small shift in frequency for both cell lines along with the negligible increases in size and zeta potential confirmed the stealth effect of PEG on protein adsorption and subsequent cellular attachment of PEGylated NLPs.^45^ Dysopsonin proteins such as albumin, the most abundant protein in plasma, along with other proteins can easily associate with the PEG carboxyl group on the surface of the NLPs, leading to a reduced NLP cellular attachment and uptake.^57^ A similar effect to that observed with Co-NLP^(PEG-5)^ was observed for NLP^(−)^. A significant decrease in the zeta potential value affected their electrostatic interactions with the cell surface, causing a low frequency shift and subsequent cellular uptake. Interestingly, although the positive liposomes had a significant decrease in their zeta potential, they remained colloidally stable and positively charged, resulting in comparable cell attachment to their bare counterpart. In addition, the protein corona may shift some of the uptake mechanism of liposomes from membrane fusion to endocytosis.^58^ This could help to offset any loss of initial particle attachment by allowing rapid internalization of the nanoparticles, probably through the presence of vitronectin and its affinity for the ανβ3 integrin, which is highly overexpressed in melanoma cells^59^ or the presence of apolipoproteins in THP1 cells, creating additional space for NLPs to be attached and internalized.^15^

In addition, QCM-D can provide insight into the viscoelastic properties of the surface through dissipation monitoring.^60–62^ Our recorded dissipation shifts mirror the frequency shifts observed for all the NLP groups in both cell types. While the dissipation shifts were not significantly different between the corona and bare PEGylated and positively charged NLPs, they were significantly different between bare and coronated negatively charged NLPs. These findings suggest that the cell monolayer is less viscoelastic after the attachment of protein corona NLPs, which caused a decreasing trend in the dissipation shift, and that the protein coronation of negatively charged NLPs had the least effect on the cell membrane viscoelasticity. This observation is supported by the literature where non-specific adsorption of negative polystyrene nanoparticles induced local gelation of lipid bilayers leading to a more rigid surface.^63^ Changes in viscoelasticity can also be explained by NLP fusion with the cell membrane, which results in cell membrane gelation.^64^ Membrane fusion has been shown for both DOTAP-based and PEGylated liposomes.^64^ In particular, the presence of PEG in PEGylated NLPs would increase the viscoelastic properties of the surface, which may explain the higher dissipation observed for NLPs^(PEG-5)^.^65^

The QCM-D data also suggest a difference in the binding kinetics of positively charged NLPs with A375 and THP1. The A375 cells appear to adsorb the nanoparticles in a linear manner but the surface of THP1 cells saturates with nanoparticles more rapidly and nonlinearly. Although there are many articles reporting on the difference in binding kinetics and cellular uptake depending on the cell type and nanoparticle properties, further QCM-D studies are required to better evaluate the capability of this technique for performing such investigations.^66,67^

Confocal microscopy and flow cytometry results confirmed the general trend in the cellular uptake observed in the QCM-D results. The main difference was related to the higher uptake in THP1 cells. It is known that nanoparticle uptake is a two-step process initiated by the non-specific attachment of nanoparticles to the cell membrane, followed by their internalization by the cells.^68,69^ These processes are influenced by the cell type due to differences in cellular function and a variation in cell-specific surface receptors, resulting in differences in nanoparticle uptake.^52^ Examples include the difference in the uptake of silica nanoparticles by HUVECs and HeLa cells or the difference in the uptake of iron oxide nanoparticles by tumor and macrophage cells.^69,70^ In our case, except for positively charged NLPs where the uptake was similar in A375 and THP1 cells, the NLP uptake was generally higher in the THP1 cells. THP1 macrophages are immortalized innate immune cells that perform phagocytosis of foreign substances in the body, and thus it is expected that they would have increased uptake when compared to the human melanoma cell line A375.^32,71^ However, the increase of NLP attachment to THP1 cells could not be fully detected by QCM-D. We believe that the first step in NLP uptake (*i.e.*, the electrostatic interaction of NLPs with the cell membrane and their subsequent internalization) was affected by the flow.^19,22,69^ When nanoparticles are statically incubated with cells, as shown by confocal microscopy and flow cytometry experiments, they can readily bind the cell surface and interact with cell membrane receptors, enabling their uptake.

The protein corona has several effects on nanoparticle–cell interactions.^72,73^ It can inhibit cellular uptake by interfering with nanoparticle–cell membrane interactions, either by altering the electrostatic interactions or by interfering with targeting ligands. The presence of various proteins in the plasma, which interact differently with nanoparticles depending on their characteristics, can also promote cellular interactions through specific proteins if the cells have the corresponding receptors.^37,39,44,68,74,75^ This has led many authors to correlate the composition of the protein corona with the cellular uptake of nanoparticles.^6,7,76^ Certain proteins, such as immunoglobulins (IgMs), which are regularly found in the protein corona, are recognized by cell receptors and can promote cellular uptake.^77^ IgM binds readily to NLPs with low surface charges, but less readily to negatively charged phospholipids.^44,78,79^ They also play an important role in opsonization and complement activation, raising the question of whether they promote the uptake of coronated nanoparticles by immune cells.^75,79^ Some studies pointed out that approximately 27% of the adsorbed proteins are functional, suggesting that the protein corona is a multilayered structure where the organization of proteins may influence the interactions with cells, explaining some of the conflicting reports on the function of immunoglobulins or other proteins in the protein corona.^80^ Our proteomic study revealed that the low PEG NLPs have increased amounts of apolipoproteins compared to the other groups, followed by positively charged NLPs, negatively charged NLPs and high PEG NLPs. Notably, Apo A-I, the major component of high-density lipoproteins, and Apo B100, the main component of low-density lipoproteins, had the largest differences in corona content.^44,81^ The high abundance of Apo B100 in the low PEGylated and positively charged NLPs may support the hypothesis derived from the TEM images that VLDL particles are present on these NLPs but further analysis is required to confirm this hypothesis. This may have also caused the increased macrophage uptake in the PEGylated NLP groups, probably due to the presence of opsonins. However, their uptake was similar in the A375 cells since they lack the corresponding receptors. Conversely, Apo A-I can be a dysopsonin conferring stealth properties, or Apo A-I and Apo B100 may be important promoters of nanoparticle uptake in cells expressing their receptors.^39,82^ Albumin was present in the protein corona of all samples, with the highest amount on the high PEG liposomes. As albumin is the most abundant protein in human plasma with both anionic and cationic sites, it can easily interact with many nanoparticles.^44^ Albumin on positive nanoparticles could enhance cellular uptake, whereas albumin on negative nanoparticles may inhibit cellular uptake, suggesting a difference in the albumin structure that could redirect the positive nanoparticles to scavenger receptors.^57^ In addition, DOTAP has been reported to induce partial protein unfolding of albumin. These literature findings may explain the high uptake of the positively charged NLPs by scavenger THP1 cells, but electrostatic interactions are likely still the dominant factor.^83^

The Co-NLP^(PEG-5)^ group had an increased number of fibrinogen chains compared to the other groups. This observation is corroborated by the presence of an enriched fibrinogen content on carboxy-functionalized nanoparticles.^84^ Fibrinogen promotes interactions with the Mac-1 receptor in THP1 cells, suggesting that the fibrinogen binding may result in a higher uptake of nanoparticles by THP1 cells.^85^ For the Co-NLP^(−)^ group, the presence of opsonins, such as immunoglobulins, likely led to a similar uptake in THP1 cells. Their presence, together with the increased cellular interaction under static conditions, acts to circumvent the reduced electrostatic interactions, resulting in a similar uptake by THP1 cells. In the A375 cells, the loss of electrostatic interactions was not compensated for by opsonins, which may have reduced the NLPs' uptake. Our proteomic analyses also showed the presence of vitronectin in positively charged NLPs. This protein has been reported to promote NLP uptake by cancer cells due to the overexpression of the ανβ3 integrin in tumour cells, which recognize vitronectin. In addition, DOTAP-containing NLPs are also reported to recruit vitronectin.^77^

## 5 Conclusion

In this study, we have demonstrated that QCM-D would be a valuable tool for monitoring the effect of the protein corona on nanoliposome–cell interactions and potentially predicting the uptake of nanoliposomes and their binding behavior to cell membranes in real time and under dynamic flow. The protein corona affected the zeta potential of the nanoparticles, neutralizing them to approximately −20 mV, and the proteins present in the corona were dependent on the physicochemical properties of the nanoparticle. By measuring the frequency and dissipation shifts on the QCM-D quartz crystal, we found that (1) the cellular uptake is influenced by the physicochemical properties of both NLPs and the protein corona, and it is cell type dependent, (2) NLP interactions with cells affect the viscoelastic properties of the cell monolayer on the sensing surface, (3) the stealthing effect of PEG resulted in very low, near to zero, frequency and dissipation shifts for PEGylated NLPs, (4) the THP1 cells have a higher uptake of NLPs regardless of the NLP properties, confirming the role of macrophages as immune scavengers, and (5) the protein corona may have a strong inhibitory effect on the interaction of NLPs with the cell membrane, hindering their uptake under dynamic flow. The lower cellular uptake observed in the QCM-D data compared to other techniques, especially after protein coronation, highlights the importance of studying nanoparticle uptake under dynamic flow conditions. Additional investigation of the QCM-D dissipation data would further elucidate the effect of NLP uptake and the protein corona on cell viscoelastic properties, as these properties play a key role in cellular uptake.

## Data availability

The authors confirm that all data presented in this manuscript will be available upon request.

## Conflicts of interest

There are no conflicts to declare.

## Supplementary Material

NA-OLF-D4NA00783B-s001
