# Supplementary material for: An investigation of the effect of the protein corona on the cellular uptake of nanoliposomes under flow conditions using quartz crystal microgravimetry with dissipation
Source: Nanoscale Adv. 2024 Oct 30;7(1):169–84. doi: 10.1039/d4na00783b (PMC11575535; doi:10.1039/d4na00783b)
Supplement: NA-007-D4NA00783B-s001 [file NA-007-D4NA00783B-s001.pdf]

Supplementary Information (SI) for Nanoscale Advances.
This journal is © The Royal Society of Chemistry 2024

Supporting Information

An Investigation Of The Fate Of Protein Corona In Nanoliposome Cellular Uptake Under Flow Via The Use Of Quartz Crystal Microgravimetry With Dissipation

Nicholas Van der Sanden[1], Radu Alexandru Paun[1], Michael Y. Yitayew[1], Oscar Boyadjian[1], Maryam Tabrizian*[1,2]

[1] Department of Biomedical Engineering, McGill University, Montreal (QC), Canada

[2] Faculty of Dental Medicine and Oral Health Sciences, McGill University, Montreal, Canada

* Corresponding Author: maryam.tabrizian@mcgill.ca

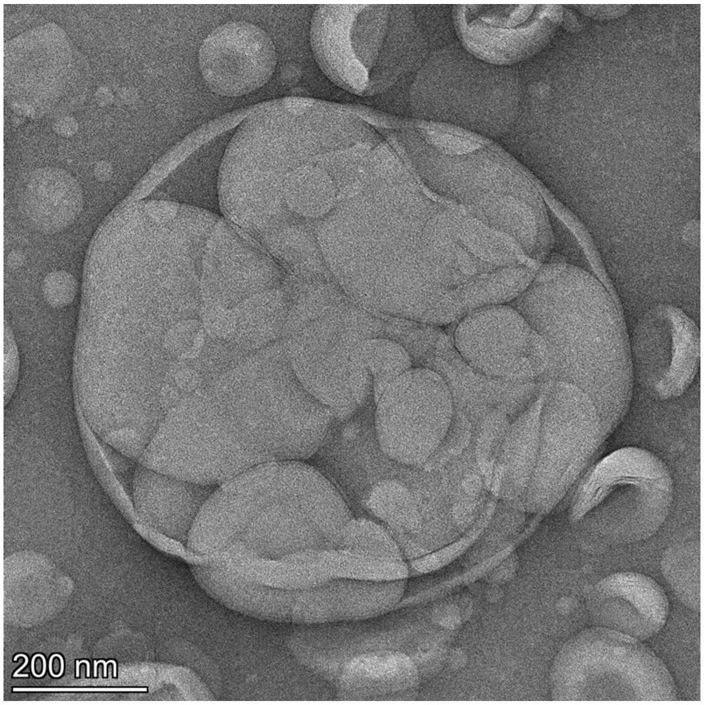

Figure S1. TEM image of low PEG NLPs showing aggregation of NLPs

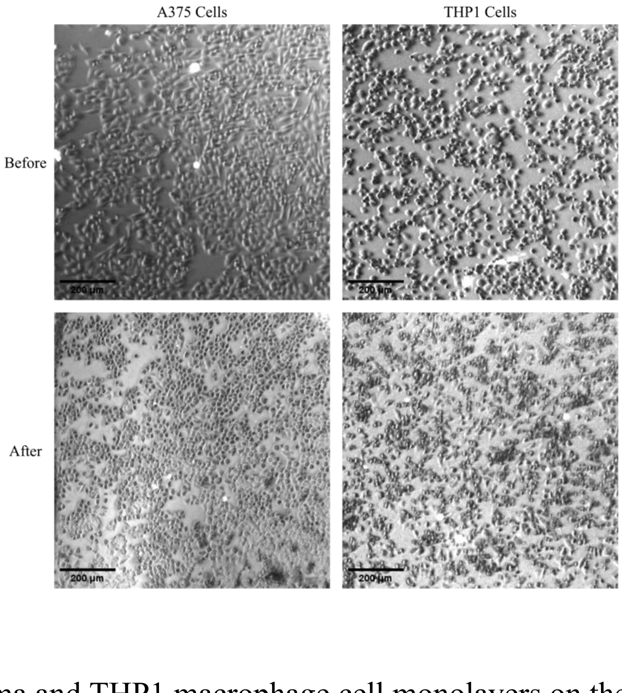

Figure S2. A375 melanoma and THP1 macrophage cell monolayers on the QCM-D crystal before and after a QCM-D experiment

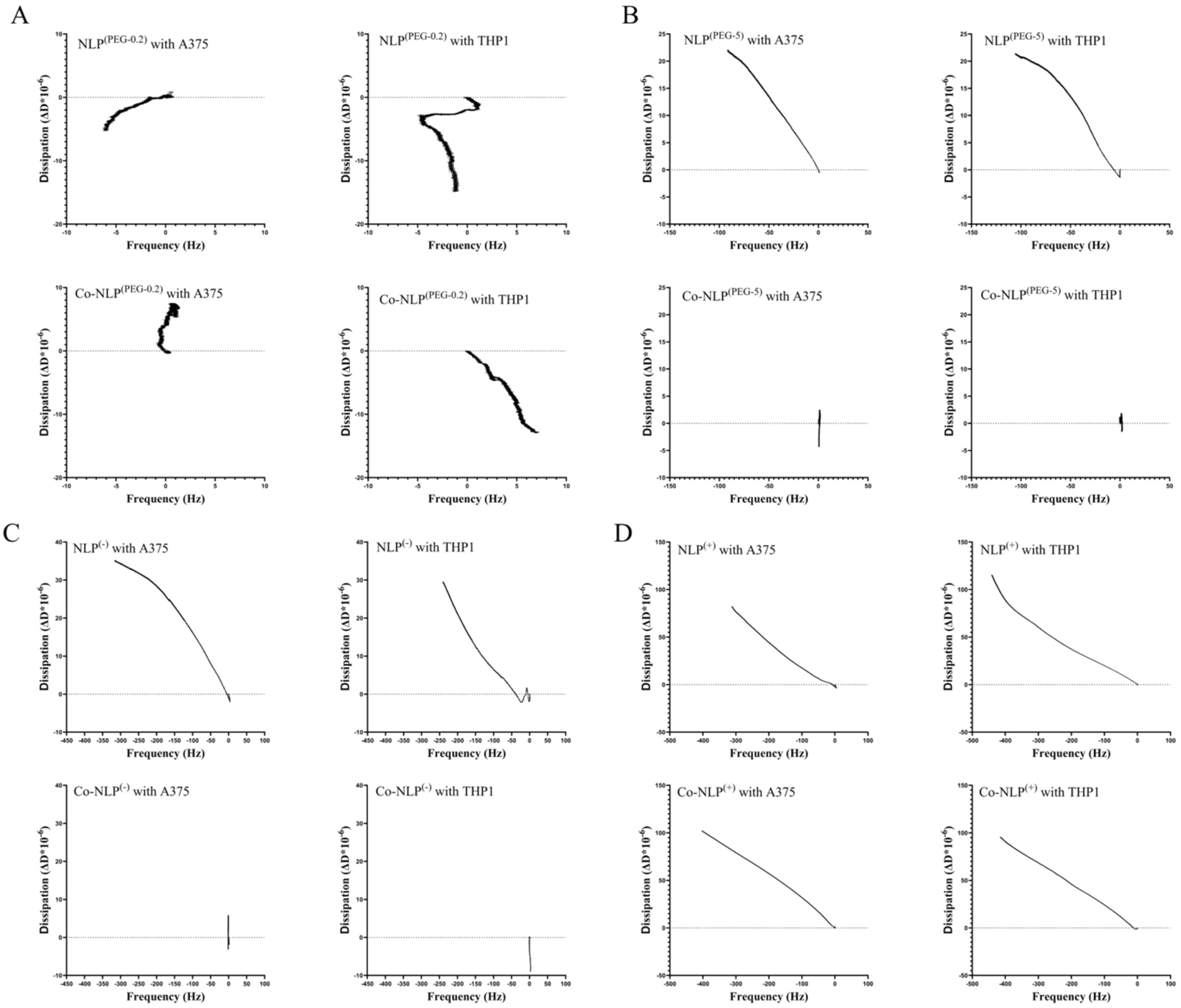

Figure S3. Mean 3rd overtone Frequency vs. Dissipation plots for all NLP formulations. Parts A and B depicts the frequency vs. dissipation plots for the low and high PEG NLPs, respectively, while parts C and D depicts the frequency vs. dissipation plots for the negatively and positively charged NLPs, respectively. In each part the first row contains the bare NLPs while the second contains the coronated NLPs, with the first column corresponding to the A375 cells and the second column the THP1 cells.

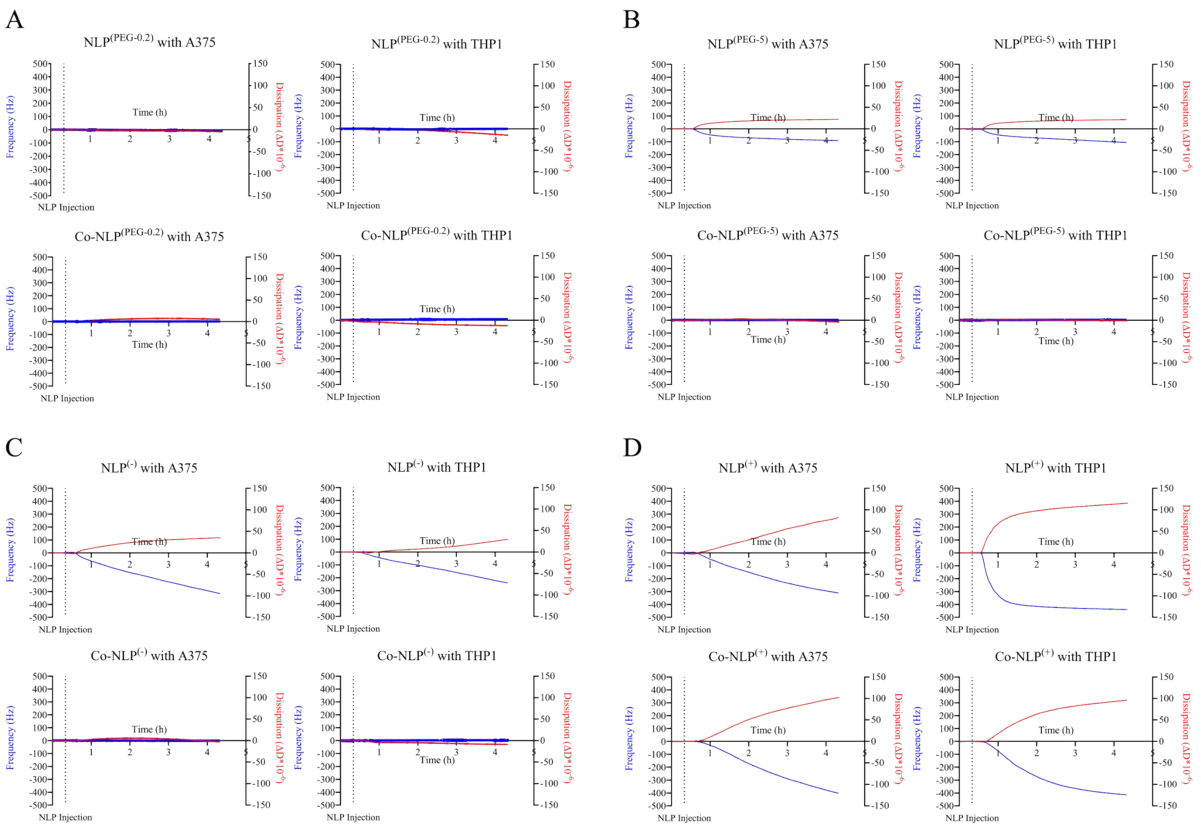

Figure S4. Mean 3rd overtone QCM-D data for all NLP formulations taken up by A375 and THP1 cells: blue lines are frequency shifts (Δf) and red lines are dissipation shifts (ΔD). Parts A and B contains all the data for the low and high PEG NLPs, respectively, while parts C and D contains all the data for the negatively and positively charged NLPs, respectively. In each part the first row contains the bare NLPs while the second contains the coronated NLPs, with the first column being A375 cells and the second column being THP1 cells. A decrease in frequency (blue lines) indicates an increase in mass on the crystal while an increase in dissipation (red lines) indicates an increase in viscoelasticity at the surface, and *vice versa*.

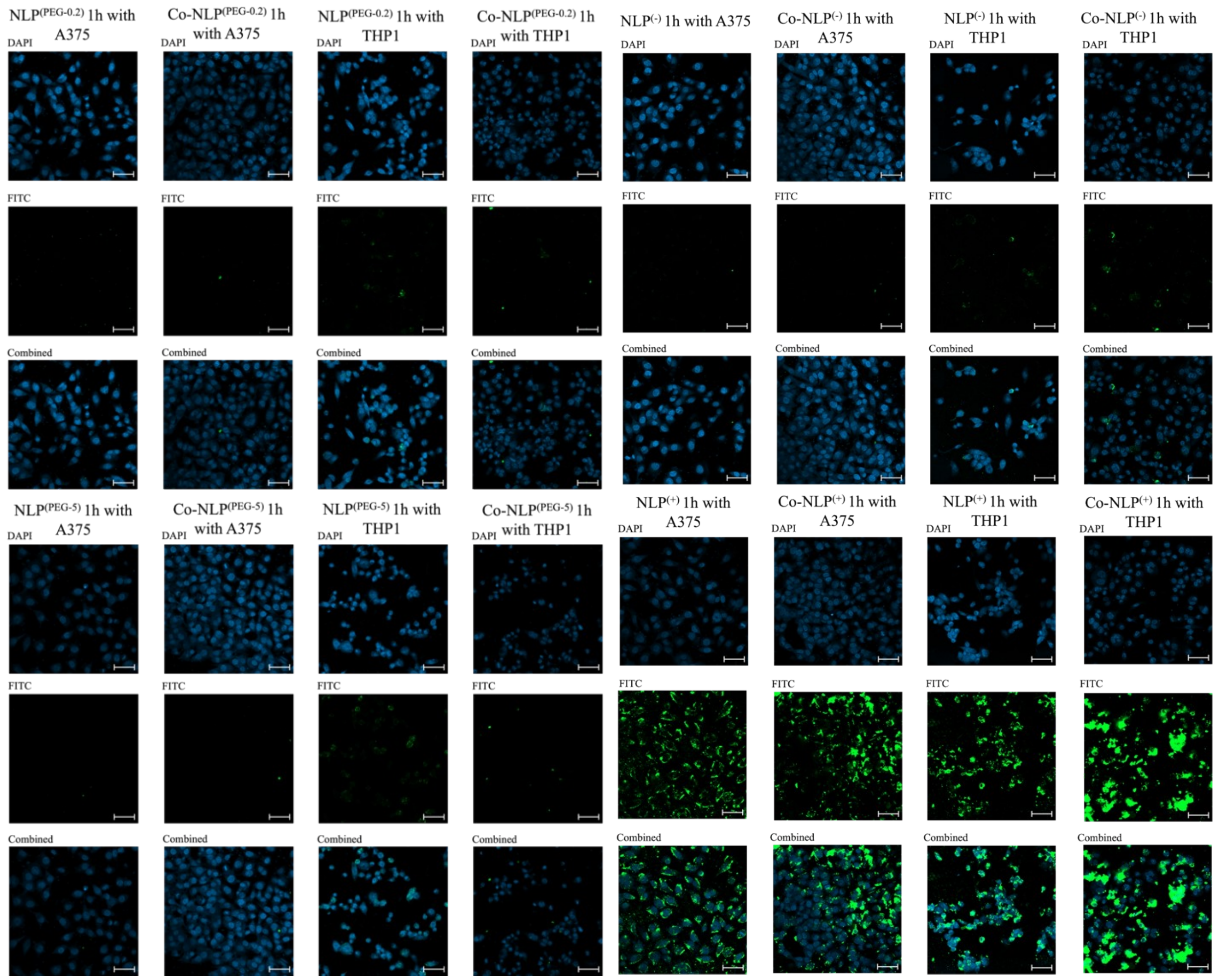

Figure S5-a. Confocal microscopy images of A375 and THP1 cells incubated with NLPs for 1 hour.

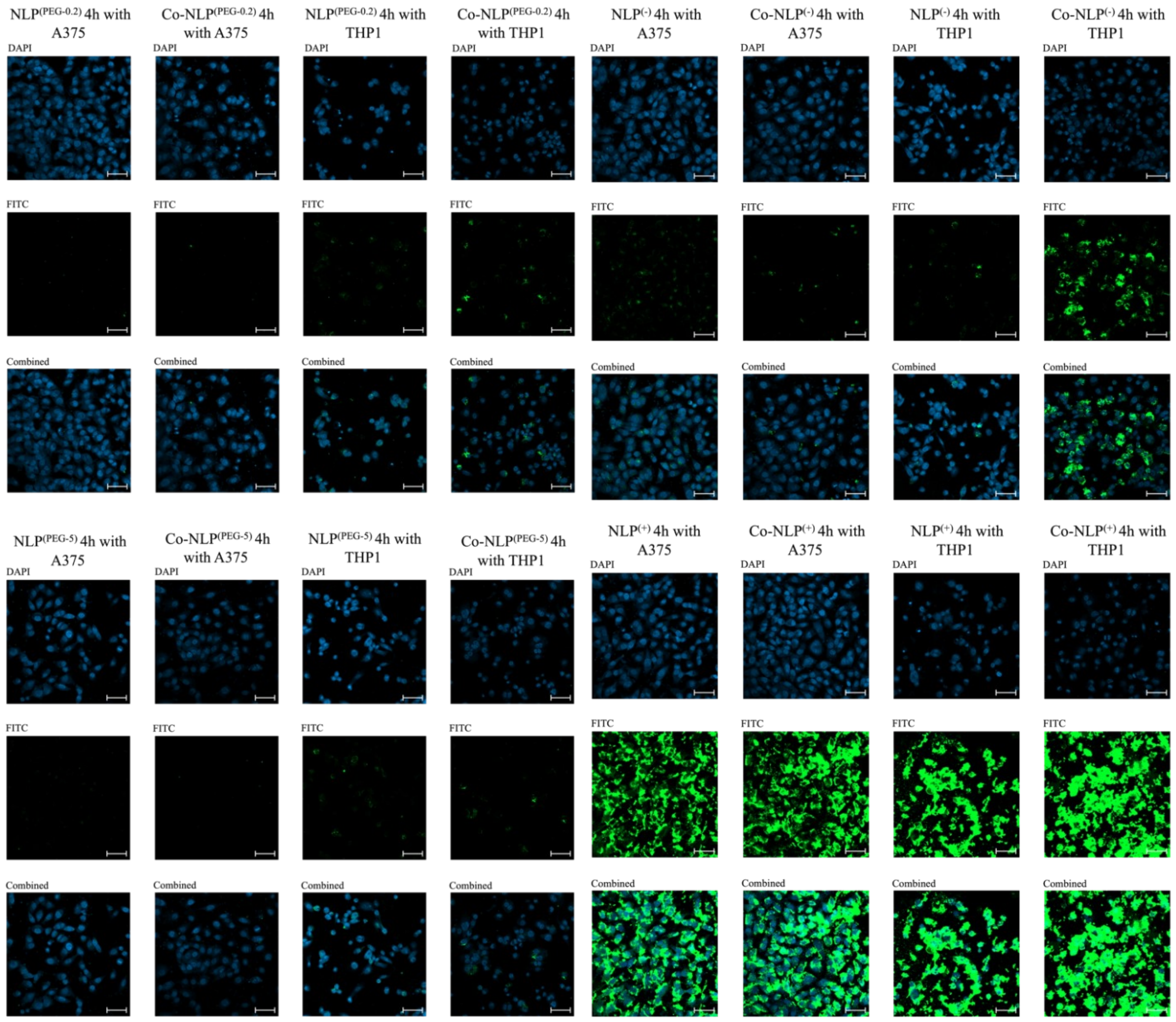

Figure S5-b. Confocal microscopy images of A375 and THP1 cells incubated with NLPs for 4 hours.

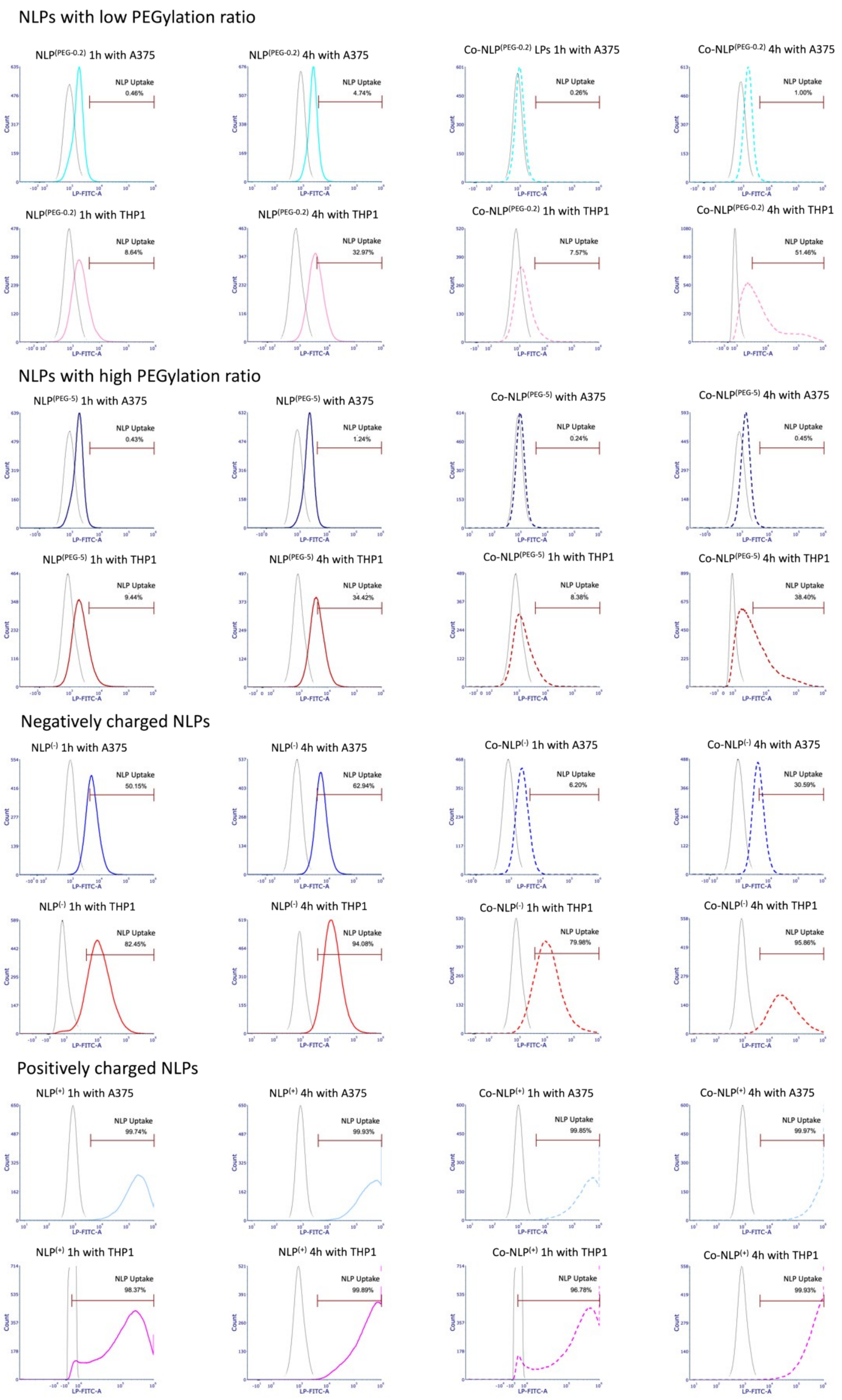

Figure S6. Representative flow cytometry histograms of A375 and THP1 cells incubated with NLPs for 1 hour and 4 hours.

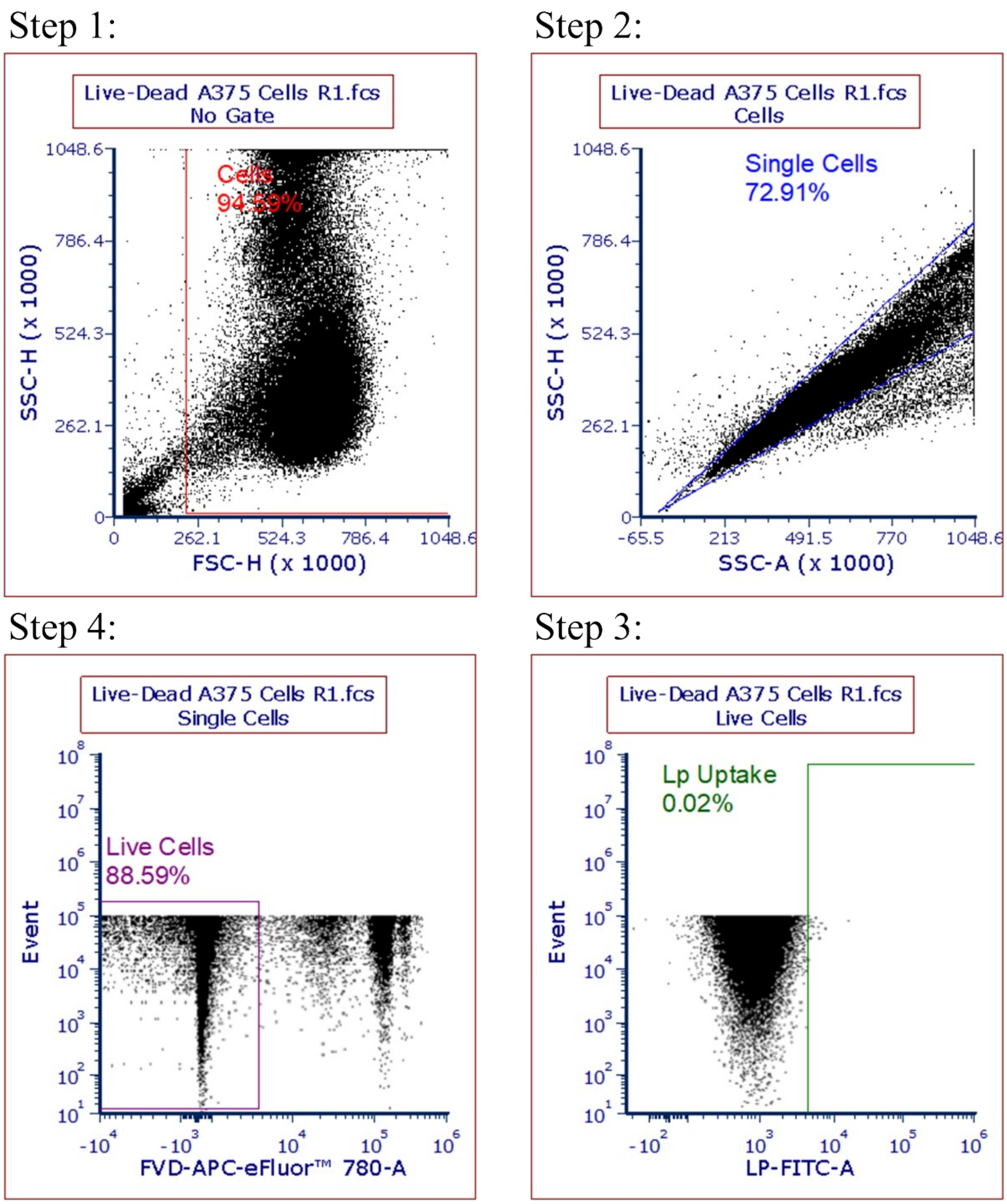

Figure S7. Example of the flow cytometry gating strategy. The first step was to remove any noise from the population, seen as the red gate in step 1. Next the single cells were selected from the cell population, seen as the blue gate in step 2. Following these dead cells were excluded from the population of single cells, done by selecting cells that were negative for the viability dye, seen as the purple gate in step 3. The final step was to select all cells with nanoliposome uptake, done by selecting cells that were positive for the FITC nanoliposomes, seen as the green gate in step 4.

Protein	Co-NLP$^{(PEG-0.2)}$	Co-NLP$^{(PEG-5)}$	Co-NLP$^{(-)}$	Co-NLP$^{(+)}$
Apolipoprotein B-100 OS=Homo sapiens OX=9606 GN=APOB PE=1 SV=2	649	-297	-289	295
Immunoglobulin heavy constant mu OS=Homo sapiens OX=9606 GN=IGHM PE=1 SV=4	108	664	759	432
Immunoglobulin mu heavy chain OS=Homo sapiens OX=9606 PE=1 SV=2	99	602	692	403
Alpha-2-macroglobulin OS=Homo sapiens OX=9606 GN=A2M PE=1 SV=3	233	454	166	224
Apolipoprotein A-I OS=Homo sapiens OX=9606 GN=APOA1 PE=1 SV=1	82	-3	-16	25
Albumin OS=Homo sapiens OX=9606 GN=ALB PE=1 SV=2	159	560	255	347
Apolipoprotein(a) OS=Homo sapiens OX=9606 GN=LPA PE=1 SV=1	67	-79	-79	-72
Trypsin OS=Sus scrofa OX=9823 PE=1 SV=1	-48	-44	-42	-50
Complement C3 OS=Homo sapiens OX=9606 GN=C3 PE=1 SV=2	97	153	83	101
Immunoglobulin gamma-1 heavy chain OS=Homo sapiens OX=9606 PE=1 SV=2	78	176	112	126
Haptoglobin OS=Homo sapiens OX=9606 GN=HP PE=1 SV=1	51	28	10	6
Immunoglobulin kappa constant OS=Homo sapiens OX=9606 GN=IGKC PE=1 SV=2	101	271	276	178
Keratin, type II cytoskeletal 1 OS=Homo sapiens OX=9606 GN=KRT1 PE=1 SV=6	0	-25	42	-11
Haptoglobin-related protein OS=Homo sapiens OX=9606 GN=HPR PE=2 SV=2	47	33	32	18
Apolipoprotein E OS=Homo sapiens OX=9606 GN=APOE PE=1 SV=1	18	-14	-8	8
Fibrinogen gamma chain OS=Homo sapiens OX=9606 GN=FGG PE=1 SV=3	25	96	29	11
Keratin, type I cytoskeletal 10 OS=Homo sapiens OX=9606 GN=KRT10 PE=1 SV=6	2	-14	60	14
Immunoglobulin kappa light chain OS=Homo sapiens OX=9606 PE=1 SV=1	88	238	252	165
Serotransferrin OS=Homo sapiens OX=9606 GN=TF PE=1 SV=3	59	125	56	78
Apolipoprotein A-IV OS=Homo sapiens OX=9606 GN=APOA4 PE=1 SV=4	29	-1	-7	24
Immunoglobulin heavy constant gamma 3 OS=Homo sapiens OX=9606 GN=IGHG3 PE=1 SV=2	36	112	81	77
Alpha-1-antitrypsin OS=Homo sapiens OX=9606 GN=SERPINA1 PE=1 SV=3	58	72	25	36
Immunoglobulin heavy constant alpha 1 OS=Homo sapiens OX=9606 GN=IGHA1 PE=1 SV=2	64	70	75	68
Immunoglobulin lambda constant 2 OS=Homo sapiens OX=9606 GN=IGLC2 PE=1 SV=1	31	78	66	47
Apolipoprotein D OS=Homo sapiens OX=9606 GN=APOD PE=1 SV=1	11	-14	-19	0
Fibrinogen beta chain OS=Homo sapiens OX=9606 GN=FGB PE=1 SV=2	28	155	54	24
Keratin, type I cytoskeletal 9 OS=Homo sapiens OX=9606 GN=KRT9 PE=1 SV=3	2	-13	29	-9
Immunoglobulin heavy constant gamma 2 OS=Homo sapiens OX=9606 GN=IGHG2 PE=1 SV=2	26	102	80	61
Keratin, type II cytoskeletal 2 epidermal OS=Homo sapiens OX=9606 GN=KRT2 PE=1 SV=2	2	-11	36	2
Fibrinogen alpha chain OS=Homo sapiens OX=9606 GN=FGA PE=1 SV=2	40	121	21	12
Albumin OS=Bos taurus OX=9913 GN=ALB PE=1 SV=4	20	19	21	48
Immunoglobulin alpha-2 heavy chain OS=Homo sapiens OX=9606 PE=1 SV=2	29	35	34	31
Immunoglobulin lambda-1 light chain OS=Homo sapiens OX=9606 PE=1 SV=1	37	76	79	59
Complement C4-B OS=Homo sapiens OX=9606 GN=C4B PE=1 SV=2	51	68	39	47
Keratin, type II cytoskeletal 5 OS=Homo sapiens OX=9606 GN=KRT5 PE=1 SV=3	-7	-12	12	-6
Ficolin-3 OS=Homo sapiens OX=9606 GN=FCN3 PE=1 SV=2	2	-9	-10	-11
Keratin, type II cytoskeletal 6A OS=Homo sapiens OX=9606 GN=KRT6A PE=1 SV=3	-8	-10	11	-6
Ceruloplasmin OS=Homo sapiens OX=9606 GN=CP PE=1 SV=1	39	33	9	20
Vimentin OS=Homo sapiens OX=9606 GN=VIM PE=1 SV=4	8	-13	-13	-5
Immunoglobulin J chain OS=Homo sapiens OX=9606 GN=JCHAIN PE=1 SV=4	4	32	35	12
Keratin, type I cytoskeletal 14 OS=Homo sapiens OX=9606 GN=KRT14 PE=1 SV=4	-3	-9	5	-5
Vitronectin OS=Homo sapiens OX=9606 GN=VTN PE=1 SV=1	12	5	-1	92
Keratin, type I cytoskeletal 16 OS=Homo sapiens OX=9606 GN=KRT16 PE=1 SV=4	-12	-9	-3	-12
Actin, cytoplasmic 2 OS=Homo sapiens OX=9606 GN=ACTG1 PE=1 SV=1	5	-11	-7	1
Apolipoprotein A-II OS=Homo sapiens OX=9606 GN=APOA2 PE=1 SV=1	14	6	4	3
Clusterin OS=Homo sapiens OX=9606 GN=CLU PE=1 SV=1	15	10	5	5
Fibronectin OS=Homo sapiens OX=9606 GN=FN1 PE=1 SV=5	27	30	2	-4
Immunoglobulin heavy variable 3-7 OS=Homo sapiens OX=9606 GN=IGHV3-7 PE=1 SV=2	0	13	16	10
Complement factor H OS=Homo sapiens OX=9606 GN=CFH PE=1 SV=4	11	28	11	10
Apolipoprotein C-I OS=Homo sapiens OX=9606 GN=APOC1 PE=1 SV=1	-1	-4	10	-2
Alpha-1-antichymotrypsin OS=Homo sapiens OX=9606 GN=SERPINA3 PE=1 SV=2	8	10	1	6
Immunoglobulin heavy constant gamma 4 OS=Homo sapiens OX=9606 GN=IGHG4 PE=1 SV=1	44	82	58	65
CD5 antigen-like OS=Homo sapiens OX=9606 GN=CD5L PE=1 SV=1	16	55	73	49
C4b-binding protein alpha chain OS=Homo sapiens OX=9606 GN=C4BPA PE=1 SV=2	13	40	30	29
Immunoglobulin kappa variable 3-20 OS=Homo sapiens OX=9606 GN=IGKV3-20 PE=1 SV=2	5	13	15	6
Hemopexin OS=Homo sapiens OX=9606 GN=HPX PE=1 SV=2	13	36	11	24
Galectin-3-binding protein OS=Homo sapiens OX=9606 GN=LGALS3BP PE=1 SV=1	12	14	8	20
Apolipoprotein L1 OS=Homo sapiens OX=9606 GN=APOL1 PE=1 SV=5	8	0	0	6
Complement factor B OS=Homo sapiens OX=9606 GN=CFB PE=1 SV=2	7	12	-1	6
Immunoglobulin heavy variable 3-30 OS=Homo sapiens OX=9606 GN=IGHV3-30 PE=1 SV=2	0	13	17	10
Apolipoprotein C-III OS=Homo sapiens OX=9606 GN=APOC3 PE=1 SV=1	0	-3	-4	-1
Immunoglobulin heavy variable 3-43D OS=Homo sapiens OX=9606 GN=IGHV3-43D PE=3 SV=1	-2	10	11	6
Inter-alpha-trypsin inhibitor heavy chain H2 OS=Homo sapiens OX=9606 GN=ITIH2 PE=1 SV=2	8	9	4	123
Antithrombin-III OS=Homo sapiens OX=9606 GN=SERPINC1 PE=1 SV=1	5	12	4	1
Annexin A2 OS=Homo sapiens OX=9606 GN=ANXA2 PE=1 SV=2	1	-5	-5	-5
Immunoglobulin kappa variable 4-1 OS=Homo sapiens OX=9606 GN=IGKV4-1 PE=1 SV=1	5	11	14	9
Immunoglobulin kappa variable 3-11 OS=Homo sapiens OX=9606 GN=IGKV3-11 PE=1 SV=1	0	7	6	6
Immunoglobulin heavy variable 3-74 OS=Homo sapiens OX=9606 GN=IGHV3-74 PE=3 SV=1	2	14	15	9
Complement C1q subcomponent subunit C OS=Homo sapiens OX=9606 GN=C1QC PE=1 SV=3	0	20	20	16
Inter-alpha-trypsin inhibitor heavy chain H4 OS=Homo sapiens OX=9606 GN=ITIH4 PE=1 SV=4	6	15	6	10
Transferrin receptor protein 1 OS=Homo sapiens OX=9606 GN=TFRC PE=1 SV=2	7	-1	3	-3
Serum amyloid P-component OS=Homo sapiens OX=9606 GN=APCS PE=1 SV=2	1	2	1	11
Kininogen-1 OS=Homo sapiens OX=9606 GN=KNG1 PE=1 SV=2	3	7	0	1
Endoplasmic reticulum chaperone BiP OS=Homo sapiens OX=9606 GN=HSPA5 PE=1 SV=2	-2	-4	-4	-4
Glyceraldehyde-3-phosphate dehydrogenase OS=Homo sapiens OX=9606 GN=GAPDH PE=1 SV=3	8	-4	-2	-1
Angiotensinogen OS=Homo sapiens OX=9606 GN=AGT PE=1 SV=1	12	5	5	11
Transthyretin OS=Homo sapiens OX=9606 GN=TTR PE=1 SV=1	13	18	9	4
Immunoglobulin kappa variable 3-15 OS=Homo sapiens OX=9606 GN=IGKV3-15 PE=1 SV=2	3	7	10	4
Immunoglobulin lambda variable 1-47 OS=Homo sapiens OX=9606 GN=IGLV1-47 PE=1 SV=2	0	7	8	5
Platelet factor 4 OS=Homo sapiens OX=9606 GN=PF4 PE=1 SV=2	2	-4	2	-4
Prothymosin alpha OS=Homo sapiens OX=9606 GN=PTMA PE=1 SV=2	-4	-4	-4	-4
Immunoglobulin lambda variable 3-9 OS=Homo sapiens OX=9606 GN=IGLV3-9 PE=3 SV=1	2	4	1	-1
Alpha-1-acid glycoprotein 2 OS=Homo sapiens OX=9606 GN=ORM2 PE=1 SV=2	8	9	8	3
Alpha-1B-glycoprotein OS=Homo sapiens OX=9606 GN=A1BG PE=1 SV=4	6	13	7	9
Hemoglobin subunit beta OS=Homo sapiens OX=9606 GN=HBB PE=1 SV=2	6	5	3	0
Apolipoprotein C-II OS=Homo sapiens OX=9606 GN=APOC2 PE=1 SV=1	8	3	1	6
Tubulin alpha-1B chain OS=Homo sapiens OX=9606 GN=TUBA1B PE=1 SV=1	5	-3	-3	-1
Tropomyosin alpha-1 chain OS=Homo sapiens OX=9606 GN=TPM1 PE=1 SV=2	-1	-3	-3	-3
Elongation factor 1-alpha 1 OS=Homo sapiens OX=9606 GN=EEF1A1 PE=1 SV=1	-2	-3	-3	-2
Serum paraoxonase/arylesterase 1 OS=Homo sapiens OX=9606 GN=PON1 PE=1 SV=3	15	11	8	19
Plasma protease C1 inhibitor OS=Homo sapiens OX=9606 GN=SERPING1 PE=1 SV=2	4	16	9	17
Apolipoprotein M OS=Homo sapiens OX=9606 GN=APOM PE=1 SV=2	3	-3	-2	1
Vitamin K-dependent protein S OS=Homo sapiens OX=9606 GN=PROS1 PE=1 SV=1	5	10	10	10
Polymeric immunoglobulin receptor OS=Homo sapiens OX=9606 GN=PIGR PE=1 SV=4	9	12	13	10
Prothrombin OS=Homo sapiens OX=9606 GN=F2 PE=1 SV=2	5	7	3	20
Immunoglobulin lambda variable 1-51 OS=Homo sapiens OX=9606 GN=IGLV1-51 PE=1 SV=2	0	8	8	6
Immunoglobulin kappa variable 2-24 OS=Homo sapiens OX=9606 GN=IGKV2-24 PE=3 SV=1	-2	0	2	-1
Apolipoprotein C-IV OS=Homo sapiens OX=9606 GN=APOC4 PE=1 SV=1	2	-2	4	-1
Complement C1q subcomponent subunit B OS=Homo sapiens OX=9606 GN=C1QB PE=1 SV=3	3	23	25	13
Immunoglobulin kappa variable 1-17 OS=Homo sapiens OX=9606 GN=IGKV1-17 PE=1 SV=2	1	3	2	2
L-lactate dehydrogenase A chain OS=Homo sapiens OX=9606 GN=LDHA PE=1 SV=2	4	-2	0	0
Histone H4 OS=Homo sapiens OX=9606 GN=H4C1 PE=1 SV=2	1	-1	1	1
Beta-2-glycoprotein 1 OS=Homo sapiens OX=9606 GN=APOH PE=1 SV=3	15	8	181	13
Immunoglobulin heavy variable 4-34 OS=Homo sapiens OX=9606 GN=IGHV4-34 PE=1 SV=2	8	19	19	16
Inter-alpha-trypsin inhibitor heavy chain H1 OS=Homo sapiens OX=9606 GN=ITIH1 PE=1 SV=3	8	10	6	106
Immunoglobulin lambda variable 9-49 OS=Homo sapiens OX=9606 GN=IGLV9-49 PE=1 SV=1	0	1	1	0
IgGFc-binding protein OS=Homo sapiens OX=9606 GN=FCGBP PE=1 SV=3	4	0	-1	-2
Transgelin OS=Homo sapiens OX=9606 GN=TAGLN PE=1 SV=4	1	-2	-2	-2
Annexin A5 OS=Homo sapiens OX=9606 GN=ANXA5 PE=1 SV=2	1	-2	-2	-2
Phosphatidylcholine-sterol acyltransferase OS=Homo sapiens OX=9606 GN=LCAT PE=1 SV=1	0	-2	-2	0
N-acetylmuramoyl-L-alanine amidase OS=Homo sapiens OX=9606 GN=PGLYRP2 PE=1 SV=1	4	-1	-1	2
Phosphoglycerate kinase 2 OS=Homo sapiens OX=9606 GN=PGK2 PE=1 SV=3	-1	-2	-2	-2

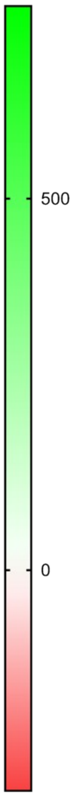

Figure S8-a: Identified proteins in the protein corona of each NLP.

	Co-NLP$^{(PEG-0.2)}$	Co-NLP$^{(PEG-5)}$	Co-NLP$^{(-)}$	Co-NLP$^{(+)}$
Complement C1r subcomponent OS=Homo sapiens OX=9606 GN=C1R PE=1 SV=2	10	28	28	33
Immunoglobulin heavy variable 5-51 OS=Homo sapiens OX=9606 GN=IGHV5-51 PE=3 SV=1	6	11	11	7
Moesin OS=Homo sapiens OX=9606 GN=MSN PE=1 SV=3	1	-2	-2	-1
Peroxiredoxin-1 OS=Homo sapiens OX=9606 GN=PRDX1 PE=1 SV=1	-1	-2	-1	-2
Pyruvate kinase PKM OS=Homo sapiens OX=9606 GN=PKM PE=1 SV=4	1	-2	-2	-1
Histidine-rich glycoprotein OS=Homo sapiens OX=9606 GN=HRG PE=1 SV=1	13	11	11	10
14-3-3 protein zeta/delta OS=Homo sapiens OX=9606 GN=YWHAZ PE=1 SV=1	-2	-2	-2	-2
Immunoglobulin kappa variable 2-30 OS=Homo sapiens OX=9606 GN=IGKV2-30 PE=3 SV=2	3	5	9	3
Alpha-1-acid glycoprotein 1 OS=Homo sapiens OX=9606 GN=ORM1 PE=1 SV=1	8	12	6	4
Complement C5 OS=Homo sapiens OX=9606 GN=C5 PE=1 SV=4	6	17	6	11
(Bos taurus) 121 kDa protein	2	7	1	2
Alpha-S1-casein OS=Bos taurus OX=9913 GN=CSN1S1 PE=1 SV=2	1	-1	0	1
Protein S100-A8 OS=Homo sapiens OX=9606 GN=S100A8 PE=1 SV=1	-1	-1	1	-1
Alpha-enolase OS=Homo sapiens OX=9606 GN=ENO1 PE=1 SV=2	6	-1	-1	0
Complement C1s subcomponent OS=Homo sapiens OX=9606 GN=C1S PE=1 SV=1	1	29	24	33
Filamin-A OS=Homo sapiens OX=9606 GN=FLNA PE=1 SV=4	2	-1	-1	-1
Plasminogen OS=Homo sapiens OX=9606 GN=PLG PE=1 SV=2	5	15	1	10
Hemoglobin subunit alpha OS=Homo sapiens OX=9606 GN=HBA1 PE=1 SV=2	1	3	3	1
Serum amyloid A-2 protein OS=Homo sapiens OX=9606 GN=SAA2 PE=1 SV=1	1	-1	-1	0
Fructose-bisphosphate aldolase A OS=Homo sapiens OX=9606 GN=ALDOA PE=1 SV=2	2	-1	-1	-1
Vitamin D-binding protein OS=Homo sapiens OX=9606 GN=GC PE=1 SV=2	3	23	9	15
Immunoglobulin lambda variable 7-43 OS=Homo sapiens OX=9606 GN=IGLV7-43 PE=3 SV=2	1	4	3	4
Immunoglobulin kappa variable 1-27 OS=Homo sapiens OX=9606 GN=IGKV1-27 PE=3 SV=1	3	13	15	8
Gelsolin OS=Homo sapiens OX=9606 GN=GSN PE=1 SV=1	9	10	5	8
Immunoglobulin heavy variable 1-8 OS=Homo sapiens OX=9606 GN=IGHV1-8 PE=3 SV=1	1	5	5	3
Immunoglobulin heavy variable 1-2 OS=Homo sapiens OX=9606 GN=IGHV1-2 PE=1 SV=2	0	3	5	1
Immunoglobulin kappa variable 1-16 OS=Homo sapiens OX=9606 GN=IGKV1-16 PE=1 SV=2	1	5	4	3
Immunoglobulin kappa variable 1D-33 OS=Homo sapiens OX=9606 GN=IGKV1D-33 PE=1 SV=2	1	5	4	2
Immunoglobulin lambda variable 3-25 OS=Homo sapiens OX=9606 GN=IGLV3-25 PE=1 SV=2	2	2	4	-1
Immunoglobulin lambda variable 3-19 OS=Homo sapiens OX=9606 GN=IGLV3-19 PE=1 SV=2	2	3	3	2
Complement component C8 beta chain OS=Homo sapiens OX=9606 GN=C8B PE=1 SV=3	5	6	1	3
Thrombospondin-1 OS=Homo sapiens OX=9606 GN=THBS1 PE=1 SV=2	2	-1	0	5
Cofilin-1 OS=Homo sapiens OX=9606 GN=CFL1 PE=1 SV=3	1	-1	-1	-1
Phosphatidylinositol-glycan-specific phospholipase D OX=9606 GN=GPLD1 PE=1 SV=3	1	-1	-1	-1
Selenoprotein P OS=Homo sapiens OX=9606 GN=SELENOP PE=1 SV=3	1	0	-1	1
C4b-binding protein beta chain OS=Homo sapiens OX=9606 GN=C4BPB PE=1 SV=1	-1	3	3	3
Serum amyloid A-4 protein OS=Homo sapiens OX=9606 GN=SAA4 PE=1 SV=2	15	7	5	10
Histone H2B type 1-K OS=Homo sapiens OX=9606 GN=H2BC12 PE=1 SV=3	-1	0	1	1
Immunoglobulin delta heavy chain OS=Homo sapiens OX=9606 PE=1 SV=1	0	10	7	6
Immunoglobulin lambda variable 2-11 OS=Homo sapiens OX=9606 GN=IGLV2-11 PE=1 SV=2	1	2	4	2
Carboxypeptidase N subunit 2 OS=Homo sapiens OX=9606 GN=CPN2 PE=1 SV=3	1	3	1	3
Protein AMBP OS=Homo sapiens OX=9606 GN=AMBP PE=1 SV=1	0	4	1	23
Prenylcysteine oxidase 1 OS=Homo sapiens OX=9606 GN=PCYOX1 PE=1 SV=3	5	-1	-1	3
Alpha-actinin-1 OS=Homo sapiens OX=9606 GN=ACTN1 PE=1 SV=2	1	-1	-1	-1
Complement factor I OS=Homo sapiens OX=9606 GN=CFI PE=1 SV=2	0	3	0	0
Kallistatin OS=Homo sapiens OX=9606 GN=SERPINA4 PE=1 SV=3	3	0	-1	2
HLA class I histocompatibility antigen, B alpha chain OX=9606 GN=HLA-B PE=1 SV=3	2	-1	-1	-1
Lipopolysaccharide-binding protein OS=Homo sapiens OX=9606 GN=LBP PE=1 SV=3	7	3	10	14
Complement C1q subcomponent subunit A OS=Homo sapiens OX=9606 GN=C1QA PE=1 SV=2	-1	5	8	4
Plasma kallikrein OS=Homo sapiens OX=9606 GN=KLKB1 PE=1 SV=1	5	11	3	4
Immunoglobulin heavy variable 3-72 OS=Homo sapiens OX=9606 GN=IGHV3-72 PE=3 SV=1	2	7	5	2
Immunoglobulin lambda variable 8-61 OS=Homo sapiens OX=9606 GN=IGLV8-61 PE=3 SV=7	2	0	2	1
Lactotransferrin OS=Homo sapiens OX=9606 GN=LTF PE=1 SV=6	0	0	2	0
Immunoglobulin lambda variable 1-40 OS=Homo sapiens OX=9606 GN=IGLV1-40 PE=1 SV=2	1	4	5	3
Histone H2A type 1 OS=Homo sapiens OX=9606 GN=H2AC11 PE=1 SV=2	1	1	4	2
Immunoglobulin heavy variable 6-1 OS=Homo sapiens OX=9606 GN=IGHV6-1 PE=3 SV=1	0	5	5	4
Protein S100-A9 OS=Homo sapiens OX=9606 GN=S100A9 PE=1 SV=1	0	0	2	0
Afamin OS=Homo sapiens OX=9606 GN=AFM PE=1 SV=1	2	4	1	3
Immunoglobulin kappa variable 1-39 OS=Homo sapiens OX=9606 GN=IGKV1-39 PE=1 SV=2	6	13	15	10
Immunoglobulin heavy variable 3-49 OS=Homo sapiens OX=9606 GN=IGHV3-49 PE=3 SV=1	4	6	6	4
Immunoglobulin lambda variable 2-18 OS=Homo sapiens OX=9606 GN=IGLV2-18 PE=3 SV=2	1	3	4	2
Coagulation factor V OS=Homo sapiens OX=9606 GN=F5 PE=1 SV=4	9	0	47	0
Alpha-2-antiplasmin OS=Homo sapiens OX=9606 GN=SERPINF2 PE=1 SV=3	6	5	1	20
Complement C4-A OS=Homo sapiens OX=9606 GN=C4A PE=1 SV=2	64	80	51	60
Pregnancy zone protein OS=Homo sapiens OX=9606 GN=PZP PE=1 SV=4	44	111	36	49
Immunoglobulin heavy variable 3-23 OS=Homo sapiens OX=9606 GN=IGHV3-23 PE=1 SV=2	7	19	22	15
Immunoglobulin kappa variable 1-8 OS=Homo sapiens OX=9606 GN=IGKV1-8 PE=3 SV=1	4	15	14	9
Ficolin-2 OS=Homo sapiens OX=9606 GN=FCN2 PE=1 SV=2	6	5	13	3
Immunoglobulin heavy variable 3-15 OS=Homo sapiens OX=9606 GN=IGHV3-15 PE=3 SV=1	2	11	10	5
Immunoglobulin lambda variable 3-10 OS=Homo sapiens OX=9606 GN=IGLV3-10 PE=3 SV=2	1	7	8	5
Immunoglobulin kappa variable 1-5 OS=Homo sapiens OX=9606 GN=IGKV1-5 PE=1 SV=2	5	7	8	6
Zinc-alpha-2-glycoprotein OS=Homo sapiens OX=9606 GN=AZGP1 PE=1 SV=2	2	7	5	3
Heparin cofactor 2 OS=Homo sapiens OX=9606 GN=SERPIND1 PE=1 SV=3	4	5	4	3
Mannan-binding lectin serine protease 1 OS=Homo sapiens OX=9606 GN=MASP1 PE=1 SV=3	2	2	4	1
Cathelicidin antimicrobial peptide OS=Homo sapiens OX=9606 GN=CAMP PE=1 SV=1	4	0	4	1
Immunoglobulin heavy variable 3-64D OS=Homo sapiens OX=9606 GN=IGHV3-64D PE=3 SV=1	0	2	4	2
Insulin-like growth factor-binding protein complex acid labile subunit OX=9606 PE=1 SV=1	5	1	3	6
Alpha-2-HS-glycoprotein OS=Homo sapiens OX=9606 GN=AHSG PE=1 SV=2	3	10	3	4
Prolactin-inducible protein OS=Homo sapiens OX=9606 GN=PIP PE=1 SV=1	0	0	3	0
Complement factor H-related protein 1 OS=Homo sapiens OX=9606 GN=CFHR1 PE=1 SV=2	0	0	3	4
Band 3 anion transport protein OS=Homo sapiens OX=9606 GN=SLC4A1 PE=1 SV=3	1	0	2	1
Complement component C9 OS=Homo sapiens OX=9606 GN=C9 PE=1 SV=2	4	5	2	7
C-reactive protein OS=Homo sapiens OX=9606 GN=CRP PE=1 SV=1	0	0	2	11
Lysozyme C OS=Homo sapiens OX=9606 GN=LYZ PE=1 SV=1	0	1	2	0
Immunoglobulin lambda variable 5-39 OS=Homo sapiens OX=9606 GN=IGLV5-39 PE=3 SV=1	1	2	2	1
Probable non-functional immunoglobulin heavy variable 3-35 OX=9606 GN=IGHV3-35 PE=1 SV=1	0	2	2	3
Cholesteryl ester transfer protein OS=Homo sapiens OX=9606 GN=CETP PE=1 SV=2	2	0	1	8
Complement component C8 alpha chain OS=Homo sapiens OX=9606 GN=C8A PE=1 SV=2	0	2	1	1
Sex hormone-binding globulin OS=Homo sapiens OX=9606 GN=SHBG PE=1 SV=2	3	1	1	0
Complement component C8 gamma chain OS=Homo sapiens OX=9606 GN=C8G PE=1 SV=3	1	2	1	1
Neutrophil defensin 1 OS=Homo sapiens OX=9606 GN=DEFA1 PE=1 SV=1	0	0	1	2
Hepatocyte growth factor activator OS=Homo sapiens OX=9606 GN=HGFAC PE=1 SV=1	0	2	1	1
Glutathione peroxidase 3 OS=Homo sapiens OX=9606 GN=GPX3 PE=1 SV=2	0	3	1	0
Coagulation factor XII OS=Homo sapiens OX=9606 GN=F12 PE=1 SV=3	0	2	0	0
Phospholipid transfer protein OS=Homo sapiens OX=9606 GN=PLTP PE=1 SV=1	6	1	0	9
Complement component C6 OS=Homo sapiens OX=9606 GN=C6 PE=1 SV=3	2	2	0	2
Leucine-rich alpha-2-glycoprotein OS=Homo sapiens OX=9606 GN=LRG1 PE=1 SV=2	4	2	0	0
Complement component C7 OS=Homo sapiens OX=9606 GN=C7 PE=1 SV=2	4	1	0	0
Pigment epithelium-derived factor OS=Homo sapiens OX=9606 GN=SERPINF1 PE=1 SV=4	3	3	0	2
Talin-1 OS=Homo sapiens OX=9606 GN=TLN1 PE=1 SV=3	3	0	0	0
Apolipoprotein F OS=Homo sapiens OX=9606 GN=APOF PE=1 SV=2	3	0	0	0
Inter-alpha-trypsin inhibitor heavy chain H3 OS=Homo sapiens OX=9606 GN=ITIH3 PE=1 SV=2	2	0	0	48
Integrin alpha-IIb OS=Homo sapiens OX=9606 GN=ITGA2B PE=1 SV=3	2	0	0	0
Complement C2 OS=Homo sapiens OX=9606 GN=C2 PE=1 SV=2	2	0	0	0
Profilin-1 OS=Homo sapiens OX=9606 GN=PFN1 PE=1 SV=2	2	0	0	0
Hyaluronan-binding protein 2 OS=Homo sapiens OX=9606 GN=HABP2 PE=1 SV=1	1	0	0	68
Lumican OS=Homo sapiens OX=9606 GN=LUM PE=1 SV=2	1	1	0	7
Cartilage oligomeric matrix protein OS=Homo sapiens OX=9606 GN=COMP PE=1 SV=2	0	0	0	4
Thrombospondin-4 OS=Homo sapiens OX=9606 GN=THBS4 PE=1 SV=2	0	0	0	4
Secreted phosphoprotein 24 OS=Homo sapiens OX=9606 GN=SPP2 PE=1 SV=1	0	0	0	2

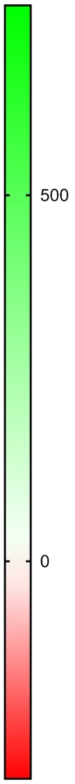

Figure S8-b: Identified proteins in the protein corona of each NLP.
